# Maternal Western diet increases inflammatory markers and decreases barrier function of offspring in *Papio anubis*

**DOI:** 10.1152/ajpgi.00342.2024

**Published:** 2025-07-10

**Authors:** Grant H. Gershner, Alena Golubkova, Cody Dalton, Camille Schlegel, Chase Calkins, Darlene N. Reuter, Megan Learner, James F. Papin, Sunam Gurung, Karen R. Jonscher, Dean A. Myers, Catherine J. Hunter

**Affiliations:** 1 Division of Pediatric Surgery, Oklahoma Children’s Hospital, Oklahoma City, Oklahoma, United States;; 2 Department of Surgery, The University of Oklahoma Health Sciences Center, Oklahoma City, Oklahoma, United States;; 3 Division of Comparative Medicine, The University of Oklahoma Health Sciences Center, Oklahoma City, Oklahoma, United States;; 4 Department of Pathology, The University of Oklahoma Health Sciences Center, Oklahoma City, Oklahoma, United States;; 5 Department of Obstetrics and Gynecology, The University of Oklahoma Health Sciences Center, Oklahoma City, Oklahoma, United States;; 6 Department of Biochemistry and Physiology, The University of Oklahoma Health Sciences Center, Oklahoma City, Oklahoma, United States

**Keywords:** *barrier function*, Papio anubis, *TransWell*, *Western diet*

## Abstract

The Western diet (WD) has been associated with various pathologies, largely due to chronic inflammatory responses triggered by insulin spikes and excess cholesterol. However, the effects of maternal WD on offspring are currently understudied. We hypothesize that maternal WD consumption in baboons induces a hyperinflammatory state in offspring, leading to compromised intestinal barrier function. Intestinal tissue was harvested from olive baboon (*Papio anubis*) 0.9 gestation fetuses and juveniles (age 2–3 yr), whose mothers were fed either a high-fat/high-sugar WD or a control diet (CD) of standard monkey chow. RNA and protein were isolated and analyzed for markers of inflammation and apoptosis. Intestinal organoids (enteroids) were generated from these bowel samples and subsequently subjected to hypoxia and LPS to simulate necrotizing enterocolitis (NEC). RNA was extracted and similarly examined for inflammatory markers and markers of apoptosis. Enteroids were plated onto TransWellTM plates to evaluate barrier function. Immunohistochemistry and immunofluorescence were used to evaluate barrier proteins. The intestinal tissue of baboon fetuses and juveniles of mothers fed a Western diet exhibited evidence of a hyperinflammatory state. Although not all cytokines reached our significance set a priori at *P* < 0.05, interleukin-8 (IL-8) and Interferon γ (IFNγ) were significantly elevated. This trend was stable across generations. Offspring from the Western diet group exhibited decreased barrier function based on transepithelial resistance measurements. Maternal consumption of a Western diet during gestation in olive baboons leads to a generalized inflammatory state and weakened intestinal barrier function in offspring, with potential long-term health implications.

## NEW & NOTEWORTHY

Our research examines a currently understudied area of the maternal/fetal relationship. This is especially pertinent, given the rampant access to high-fat/high-sugar foods in the Western diet. It is also noteworthy due to the findings of decreased barrier function among those in the Western diet group. This decreased function and hyperinflammatory state can prime neonates for various pathologies, such as necrotizing enterocolitis.

## INTRODUCTION

Obesity and dietary choices have been studied as potential triggers and contributors to common medical problems. For example, correlations with increased cancer risk (1), worse surgical outcomes (2), and exacerbating common medical conditions (3, 4) have all been described. This is also true in pregnancy. Pregnant individuals with a body mass index (BMI) > 30 (obese) or excessive weight gain (15–25 lbs. overweight, 15 lbs. obese) have a higher risk for gestational diabetes, hypertension, preeclampsia, and birth/labor complications (5). Maternal obesity increases maternal complications but also conveys increased morbidity and potential lifelong implications for the fetus. These, acutely, include low activity, pulse, grimace, appearance, respiration (APGAR) scores; premature birth; macrosomia; and neurologic injury, as well as increased risk of obesity and other chronic metabolic syndromes (6).

Pregnancy is associated with a metainflammatory state that is heightened by obesity, with primary increases in circulating interleukin 6 (IL-6) and C-reactive protein (CRP) (1, 7, 8). Adipose tissue, primarily abdominal fat, is inflamed in the obese state and contributes to the increased systemic levels of IL-6 observed in obesity (9). Plasma CRP is also elevated (10); however, this cytokine is primarily of hepatic origin (11). Both obesity and a Western diet are associated with an increase in activated monocytes, which contributes to adipose tissue macrophage populations and the associated inflammation (12, 13). This subsequently results in the systemic activation of inflammatory pathways, namely c-Jun N-terminal kinase (JNK), nuclear factor κB (NF-κB), and protein kinase R (14). Inflammation also is partly due to the Western diet (WD). Western diets are calorically dense, low in fiber, and highly processed (15). Western diets also have high glycemic indexes (GIs) (16) and are high in saturated fats as well as cholesterol, the latter being a potent activator of monocytes (17).

A review of the literature shows that although the effects of this chronic, low-grade inflammatory state driven by obesity and WD have been examined in pregnancy (6), most studies examining direct effects on fetuses have used murine models (18–20). Although some studies associate maternal pregravid obesity with the risk of necrotizing enterocolitis (NEC) in neonates, the link between maternal obesity and NEC remains equivocal, most likely reflecting the wide range of maternal comorbidities and variability in maternal diet in human pregnancy (21). Nonetheless, WDs do contribute significantly to the ongoing obesity epidemic. In this study, we examine the inflammatory state of intestinal tissue and organoids harvested from late gestation (G) fetuses and 2- to 3-yr-old juveniles obtained from nonhuman primate (NHP; Olive baboon, *Papio anubis*) dams exposed to a WD high in saturated fats, simple sugars, supplemented with a high-fructose drink starting minimally 3 mo before breeding then over the course of gestation through lactation. We hypothesize that intestinal tissue obtained from term gestation fetuses and 2- to 3-yr-old juveniles from dams fed a Western (high fat, simple sugar) diet will show higher levels of inflammatory markers.

## MATERIALS AND METHODS

### Animal Model

All experiments involving baboons adhered to guidelines outlined by the Animal Welfare Act for the housing and care of laboratory animals and the US National Institutes of Health Office of Laboratory Animal Welfare Public Health Service Policy on Humane Care and Use of Laboratory Animals. Environmental enrichment was provided to all animals. The research was conducted with approval from the University of Oklahoma Health Sciences Center Institutional Animal Care and Use Committee (IACUC) under Protocol No. 302043#22–025-AH.

Adult, nulliparous baboon (*Papio anubis*) dams (aged 5–10 yr, *n* = 21) were equally randomized, with consideration for social status, into two diet cohorts. Dams were housed in diet groups (5 or 6 per corral) for the study’s duration. The dams that underwent cesarean section (CS) were previously described in detail, including systemic metabolic parameters, inflammation, and anthropometric measurements (22).

Dams were either fed a diet of standard baboon chow [control diet (CD); 5045, Purina LabDiets, St. Louis, MO; 30.3% calories from protein,13.2% from fat, 56.5% from carbohydrates], or high-saturated fat, high-sugar diet [“Western style diet” (WD); “TAD” Primate diet, 5L0P, Purina; 18.3% calories from protein, 36.3% of calories from fat, 45.4% from carbohydrates]. The WD-fed dams had ad libitum access to a high-fructose (100 g/L) beverage (Kool-Aid). Diets were otherwise matched in micro- and macronutritional content. All dams received daily dietary enrichment (fruit and peanuts).

After an initial 3-mo WD acclimation period and collection of baseline samples, dams (CD, *n* = 11; WD, *n* = 10) were bred to males previously fed CD but were on the WD for the duration of the breeding period when housed with the WD-fed dams. Blood, fecal samples, and anthropometric measurements (body weight, skin folds, length, and girth) were obtained under Ketamine sedation before the study start, after the 3-mo pre-feeding period before breeding at 0.6 gestation (G) and for the CS dams, 0.9 G. At 0.6 gestation, baboons were fasted overnight, and an intravenous glucose tolerance test (IVGTT) was performed under ketamine sedation. Maternal blood samples (plasma and serum) were collected at 0.6 and 0.9 gestation (at the time of CS). At 0.9 gestation, dams were anesthetized, and fetuses were delivered via CS. Fetal and placental weights were obtained, and tissue samples were processed for histology or frozen on dry ice and stored at −80°C for subsequent analyses. Another cohort for each diet group was allowed to deliver and nurse the young until they were weaned. After weaning, juveniles were socially group-housed and transitioned to the CD. Juveniles were euthanized, and tissues were obtained at 2.5–3 yr of age. In total, 11 fetuses (male: 7, female: 4) and 9 juveniles (male: 2, female: 7) were harvested.

### Bowel Sample Collection

Ileal tissue samples were obtained from baboon fetuses (*n* = 11) and juveniles (aged 2–3 yr, *n* = 9) during necropsy. Samples were either snap-frozen or washed in Dulbecco’s phosphate-buffered saline (DPBS, Sigma Life Science, #D8573) and either processed into enteroids or maintained in Roswell Park Memorial Institute (RPMI) media before processing to enteroid cultures within 24 h of necropsy.

### Harvest and Culture of Enteroids

Crypt isolation and enteroid processing were carried out as previously described by our laboratory (23). Primary intestinal crypt cultures (enteroids, small bowel organoids) were isolated from ileum and maintained in Matrigel (Corning, CB-40230C), initially with 50% LWRN/Human Minigut media then subsequently transitioned to IntestiCult Organoid Growth Media (StemCell technologies, 100–0191). If grown in LRWN/Human Minigut media, the media is supplemented with 50 ng/mL epidermal growth factor (Millipore Sigma, #GF144, Burlington, MA), 1 mM *N*-acetylcysteine (Millipore Sigma, #A9165–5G, Burlington, MA), 500 nM A-83–01 (R&D Systems, #2939/10, Minneapolis, MN), 10 μM SB202190 (Millipore Sigma, #S7067–5 MG, Burlington, MA), 10 mM nicotinamide (Millipore Sigma, #N0636–100G, Burlington, MA), and 10 nM [leu] 15-gastrin 1 (Millipore Sigma, #G9145–0.1 MG). Cultures were maintained with media changes every 2 days and passaging every 6 to 7 days, depending on growth and enteroid health. Experiments were initiated when enteroids had reached maturity between passages 4–10. Enteroid maturity was assessed at 7 days from the last passage by microscopic visualization looking for budding (invaginations on the enteroid exterior membrane).

### NEC Induction

Enteroid NEC was induced in CD or WD mature enteroids as described previously (23). Enteroids were plated onto two separate 24-well plates, each with nine wells of CD or WD. One plate underwent media changes as per our typical protocol. The other was treated with 100 μg/mL of lipopolysaccharide (LPS, Sigma-Aldrich, L2630; isolated from *Escherichia coli* O111:B4), placed in an isolation chamber, and was subjected to hypoxia (1% O_2_, 5% CO_2_, 94% N_2_) for 24 h via a modular incubator chamber. Enteroids were subsequently harvested expeditiously to limit reoxygenation.

### Total RNA Isolation and Quantitative Reverse Transcription PCR

Snap-frozen ileal tissues were homogenized using a handheld rotor-stator homogenizer and RNA was isolated following the manufacturer’s instructions using either the Invitrogen TRIzol Reagent (15596026; Life Technologies, Carlsbad, CA) or the Quiagen RNEasy Kit (74104; Quiagen, Hilden, Germany) and quantified using a NanoDrop Lite spectrophotometer (Thermo Scientific, Waltham, MA).

cDNA was generated via reverse transcription to 0.5–2 μg using a high-capacity cDNA reverse transcription kit (Applied Biosystems, #4374966, Waltham, MA). Real-time PCR was then done and analyzed using the delta-delta CT method. The genes examined were interleukin *1β (IL-1β), IL-8, IL-10, IL-12, IL-18, IL-23*, interferon γ (*IFNγ*), tumor necrosis factor α (*TNFα*), Toll-like receptor 4 (*TLR4*), caspase 3, caspase 8, Kiel 67 (*K*_*i*_-*67*), and proliferating cell nuclear antigen (*PCNA*). A list of these genes and their related primers can be found in Table 1. All gene expression was normalized against the housekeeping gene *GAPDH*. PCR was carried out using the CFX Opus 96 system and iQ SYBR Green Supermix (Biorad, Hercules, CA) with 4 ng of cDNA template and a final primer concentration of 0.5 μM.

### TransWell Two-Dimensional Culturing and Transepithelial Electrical Resistance Measurement

Mature enteroids were plated onto TransWell semi-permeable plates (3413, Corning Life Sciences, Corning, NY). This was done by first mixing Matrigel glomerular filtration rate (GFR) basement membrane (BMM, 356231, Fischer Scientific, Hampton, NH) with DMEM/F12. Plates were incubated for 2 h to allow for the basement membrane to adhere. Enteroid cultures were processed similarly to normal passaging. The BMM mixture was then removed, and enteroids were plated 1 dome to 1 well. Enteroids were maintained with Human IntestiCult Organoid Growth Media (06010, StemCell Technologies, Vancouver, BC, Canada) in both the upper and lower chambers (200 and 600 μL, respectively). Cells were then transitioned to Human IntestiCult Organoid Differentiation Media (100–0214; StemCell Technologies, Vancouver, BC, Canada) when deemed appropriate [determined after visual confluence and transepithelial electrical resistance (TEER) measurement plateau]. Wells were visually inspected using a microscope every 2–3 days and checked for confluence, and media was changed. TEER measurements were also obtained before the media was changed. TEER measurements were carried out using an epithelial voltohmmeter (EVOM-EVM-MT-03–01 and EVOM 2-EVOM2, World Precision Instruments, Sarasota, FL).

### Immunofluorescent Staining

Baboon fetal and juvenile intestine samples were fixed in 10% formaldehyde, embedded in paraffin, and sectioned for histological analysis.

Apoptosis was assessed using the ApoTag Red In Situ Apoptosis Detection Kit (S7165, Chemicon, Rolling Meadows, IL) following the manufacturer’s instructions for deparaffinizing, pretreating, equilibrating, and staining. Fluoroshield with DAPI (Sigma Aldrich, F6057–20ML) was used to mount sections and stain nuclei. Images were acquired at ×20 magnification with an ECHO Revolution microscope (ECHO, San Diego, CA) equipped with a Texas Red filter for viewing rhodamine fluorescence.

TransWell membranes coated with enteroid two-dimensional (2-D) cultures were stained using immunofluorescence to examine barrier proteins. Membranes were washed twice with warm phosphate-buffered saline (PBS) and then fixed with 2% paraformaldehyde for 1 h at room temperature. Following fixation, membranes were washed four times with PBS and then permeabilized with 0.1% Triton in PBS. Membranes were excised and transferred to a 24-well plates. Membranes were blocked with 5% normal goat serum PBS + Tween (PBST, 0.05% Tween, Sigma Aldrich, P9416) for 1 h and then incubated overnight with claudin-1 (Abcam, ab211737) at a 1:1,000 dilution. The following morning, membranes were washed with PBST four times and then incubated with a secondary antibody (A11034, Alexa Fluor 488 goat anti-rabbit) for 1 h in the dark. After incubation, membranes were washed four more times with PBS and then mounted on a slide using Fluoroshield with DAPI.

### Immunohistochemical Staining

Tissues were obtained and fixed in 10% neutral-buffered formalin, embedded in paraffin, and sectioned in 5-μm sections for routine staining. Sections were deparaffinized, rehydrated, and washed in Tris-buffered saline (TBS). Slides were processed for immunohistochemistry (IHC) using ImmPRESS Excel Amplified Polymer, Peroxidase (anti-rabbit IgG, Cat. No. MP-7601, Vector Laboratories, Newark, CA). Antigen retrieval was accomplished with pH 6 citrate antigen unmasking solution (Cat. No. H-3300) or antigen unmasking solution, Tris based (Cat. No. H-3301, Vector Laboratories Inc., Newark, CA), by 20 min in a steamer followed by 30 min cooling at room temperature. Sections were treated with a peroxidase-blocking reagent (Bloxall, Cat. No. SP-6000, Vector Laboratories, Inc., Newark, CA) to inhibit endogenous peroxidase activity, followed by 2.5% normal horse serum-blocking reagent to inhibit nonspecific binding. Appropriate washes were in TBS. Tissue sections were incubated in humidified chambers with E-cadherin (24E10) rabbit mAB (1:100 dilution, Cat. No. 3195, Cell Signaling, Danvers, MA), zona occludins 1 (ZO-1, D7D12) rabbit mAB (1:200 dilution, Cat. No. 8193, Danvers, MA), or NF-κB p65 (ab16502) rabbit mAB (1:1,000 dilution, Abcam, Cambridge, MA). Following 60 min of incubation at room temperature, sections were washed in TBS, and reagents were applied according to the manufacturer’s directions. Slides were incubated with NovaRed (Vector Laboratories, Inc., Newark, CA) chromogen for visualization. Counterstaining was carried out with hematoxylin QS nuclear counterstain (Vector laboratories, Newark, CA).

### Statistical Analysis

Comparative statistical analysis between groups was carried out using either a one-way analysis of variance (ANOVA) or an unpaired Student’s *t* test using GraphPad Prism Software (v. 10.0.0). Statistical significance was set at *P* < 0.05.

## RESULTS

### Baboon Dam Anthropometrics

Previously, we have shown that dams exposed to WD/high fructose for 386.5 ± 39 days (*n* = 5 or 6 per group) showed no significant difference in adiposity compared with CD, determined by differences in maternal body weight or sum of skin fold (SSF) thickness (22). In the current study, we increased our cohort to include dams that delivered offspring, adding seven CD- and three WD-fed dams.

**Figure 1.**
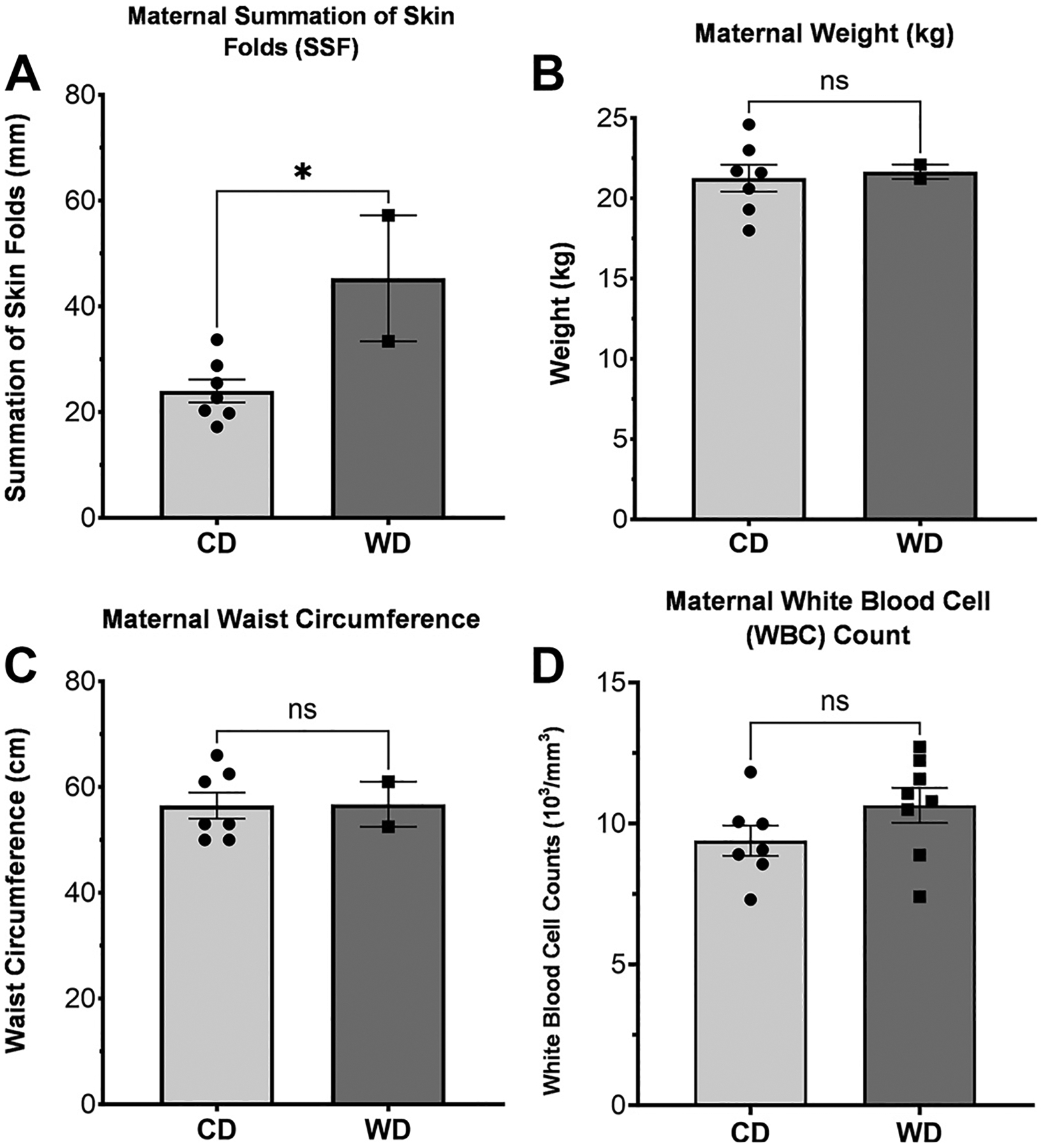
Maternal anthropometric measurements. Several forms of anthropometric data from the baboon dams, including summation of skin folds (*A*), body weight (*B*), waist circumference (*C*), and white blood cell counts (*D*). Of these, the summation of skin folds was found to be significantly different (*P* = 0.015), whereas all others were not (weight *P* = 0.819; waist *P* = 0.963; WBC *P* = 0.155). Error bars represent means ± SE. ns, not significant. * indicates significance.

Consistent with the previous findings, dams showed no significant change in weight and waist circumference between CD and WD (*P* = 0.819 and *P* = 0.963, respectively; Fig. 1, B and C); however, SSF was significantly increased in WD in our larger cohort (*P* = 0.0150; Fig. 1A). These findings are consistent with the previous reports using a similar diet and baboon model, confirming that a minimum of 9 mo to ~3 yr of WD feeding is needed for dams to attain an obese state (24, 25). Dams showed a slight increase in white blood cell counts, but this was not statistically significant (average ± CD, *P* = 0.1550).

### Inflammatory Markers are Increased in Fetuses and Juveniles Exposed to Maternal Western Diet

**Figure 2.**
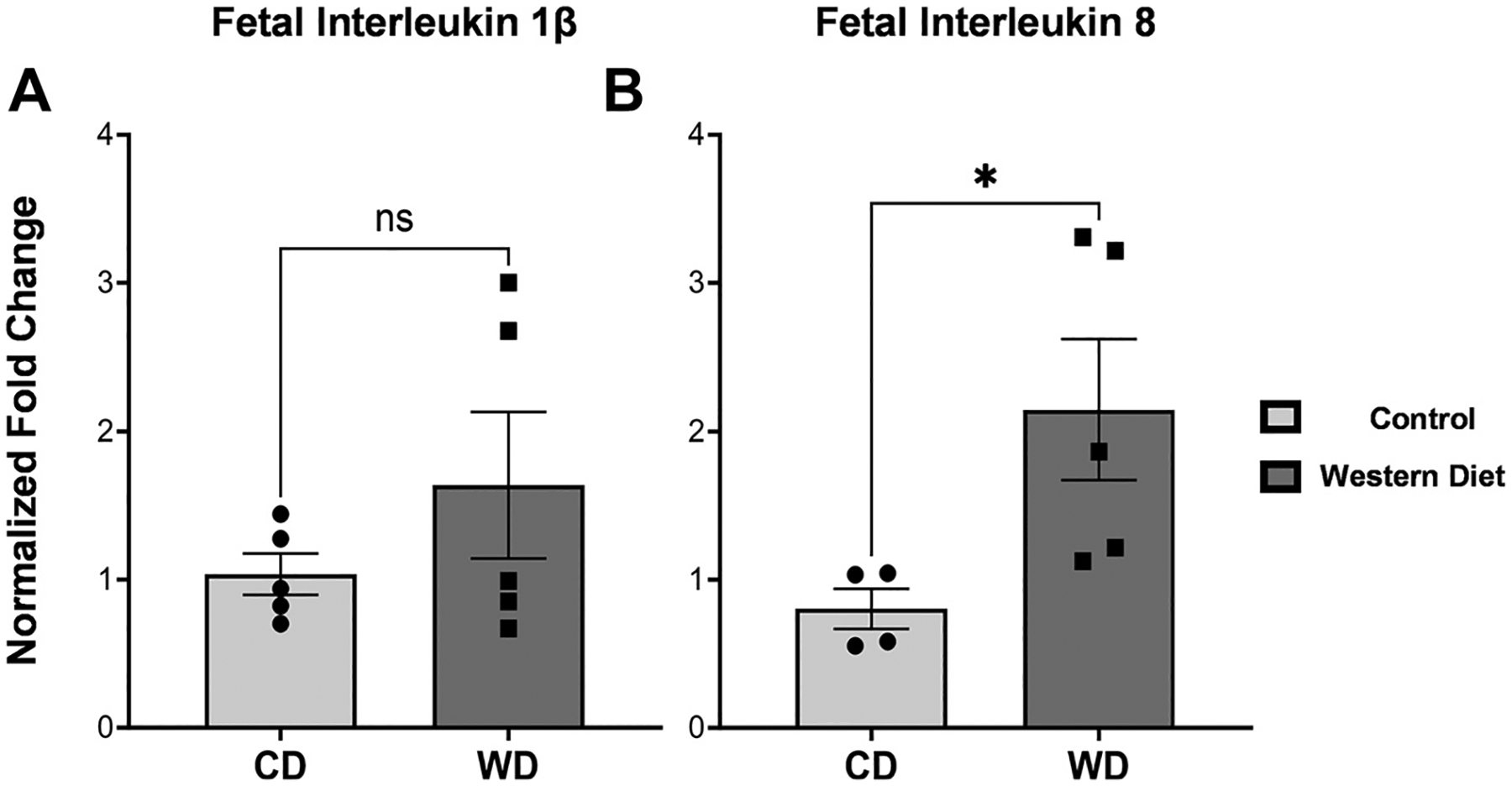
*A* and *B*: expression of interleukin-1β *(IL-1β)* and interleukin-8 in fetal baboon tissue via RT-qPCR. In fetal baboon tissue, there was a significant elevation of *IL-8* (*P* = 0.045), and a decreasing trend in *IL-1β* (*P* = 0.403). Error bars represent means ± SE. ns, not significant. * indicates significance.

Previous published work from this group has shown a significant increase in systemic inflammation in dams, therefore we sought to determine whether the intestines also exhibited a proinflammatory phenotype as a result of maternal WD exposure and whether the phenotype persisted in offspring (22). In fetuses, there were no significant differences in *IL-1β* (*P* = 0.4) expression. Levels of *IL-8* were significantly elevated (*P* = 0.0045). These elevated levels of *IL-8* mRNA suggest that the maternal WD environment did impact fetuses (Fig. 2). In summary, fetal ileum from WD dams had significantly higher levels of *IL-8*.

**Figure 3.**
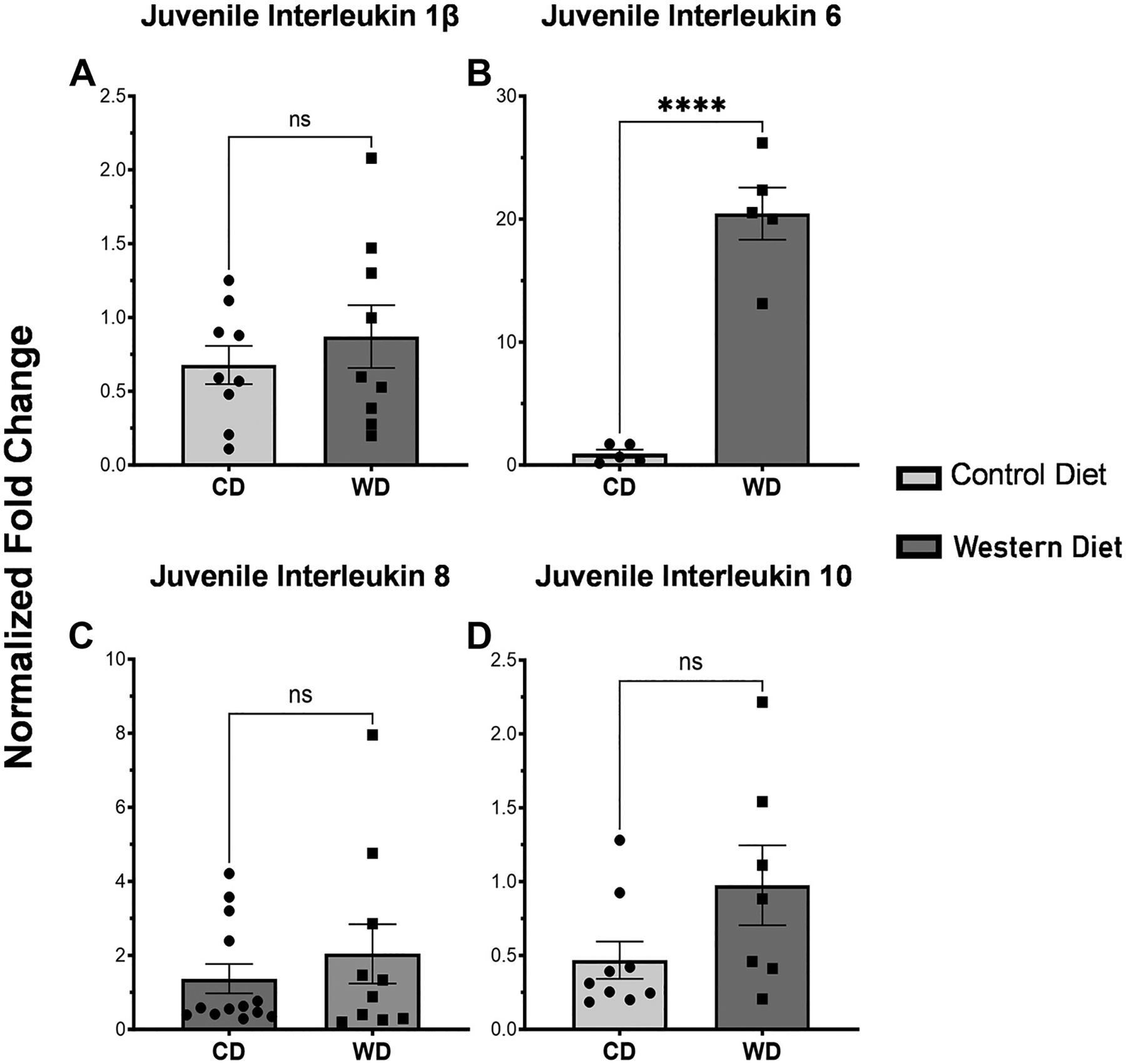
Expression of interleukin-1β (*IL-1β*), interleukin-6 (*IL-6*), interleukin-8 (*IL-8*), and interleukin-10 (*IL-10*) in juvenile baboon tissue via RTqPCR. In juvenile baboon intestinal tissue, there was a trend of elevated inflammatory markers for *IL-1β* (*A*), *IL-8* (*C*), and *IL-10* (*D*). These were not statistically significant (*IL-1β P* = 0.450, *IL-8 P* = 0.429, *IL-10 P* = 0.0878). *B*: expression of *IL-6* was significantly elevated (*P* < 0.0001). Error bars represent means ± SE. ns, not significant. **** indicates high signficance *P* < 0.0001.

**Figure 4.**
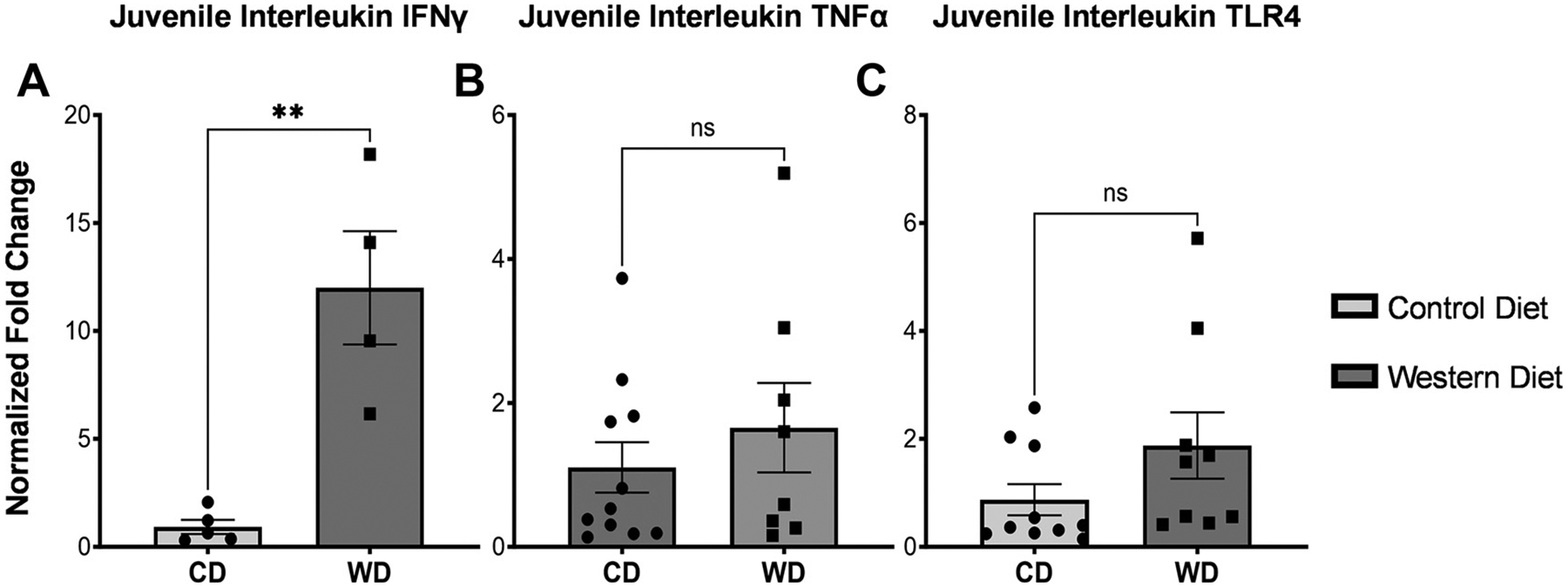
Expression of *IFN-γ, TNF-α*, and Toll-like receptor 4 (*TLR4*) in juvenile baboon tissue via RTqPCR. In juvenile baboon tissue, there was a trend to elevated inflammatory markers for *TNFα* (*B*) and *TLR4* (*C*). *IFNγ* was significantly elevated (*P* = 0.0021) (*A*), whereas *TNFα* and *TLR4* were both elevated in the Western diet group but not significantly so (*TNFα P* = 0.420, *TLR4 P* = 0.1449). Error bars represent means ± SE. ns, not significant. ** indicates significance.

Ileum from baboon juveniles showed no differences for *IL-1β* (*P* = 0.4496) and *IL-8* (*P* = 0.429) mRNA between WD versus CD dams (Fig. 3, A and C). *IL-6* mRNA was significantly increased in WD juvenile ileum (*P* < 0.0001), and *IL-10* mRNA was also not increased (*P* = 0.088) when compared with juveniles from CD dams (Fig. 3, B and D). There were no sex-specific differences (Table 2). There was significantly increased expression of *IFNγ* mRNA (*P* = 0.0021; Fig. 4) in baboon juveniles from WD dams compared with CD dams. Neither *TNFα* nor *TLR4* mRNA was altered by maternal diet (*P* = 0.4201, *P* = 0.1449, respectively; Fig. 4). In short, juvenile ileum from WD dams had significantly higher levels of *IL-6* and *IFNγ*.

### NF-κB Pathway Inflammation is not Significantly Increased in Fetuses or Juveniles Exposed to Maternal Western Diet

**Figure 5.**
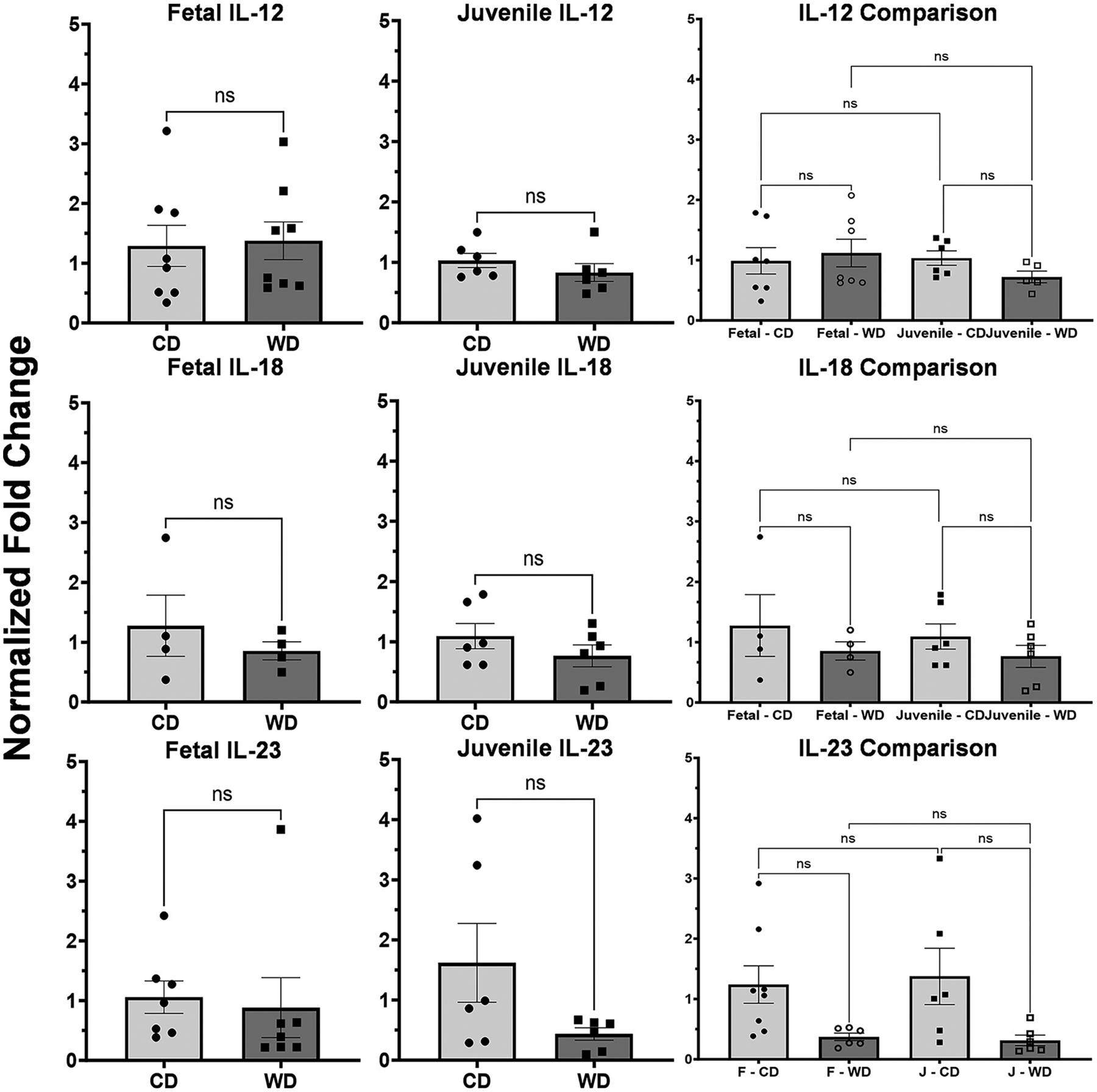
Markers of *NF*-*κB* activation are not significantly different in Western or control diet fetuses and juveniles—levels of *IL-12, IL-18*, and *IL-23* were assessed as markers of *NF*-*κB* pathway activation. *IL-12* showed no significant differences in fetuses or juveniles (*P* = 0.858 and *P* = 0.3163, respectively). There was no significant difference between juveniles or fetuses (from L to R: *P* = 0.956, *P* = 0.998, *P* = 0.505, and *P* = 0.706, respectively). *IL-18* showed no significant differences in fetuses or juveniles (*P* = 0.461 and *P* = 0.263, respectively). There was no significant difference between juveniles or fetuses (from L to R: *P* = 0.755, *P* = 0.964, *P* = 0.995, and *P* = 0.777, respectively). *IL-23* showed no significant differences in fetuses or juveniles (*P* = 0.763 and *P* = 0.104, respectively). There was no significant difference between juveniles or fetuses (from L to R: *P* = 0.169, *P* = 0.987, *P* = 0.999, and *P* = 0.095, respectively). ns, not significant.

To determine the effects of the WD on other inflammatory pathways, we examined *IL-12, IL-18*, and *IL-23*. Of these, none were significantly elevated in fetuses (*P* = 0.856, *P* = 0.461, and *P* = 0.763, respectively) or juveniles (*P* = 0.316, *P* = 0.263, and *P* = 0.104, respectively). There were no significant differences between fetal and juvenile levels (Fig. 5).

**Figure 6.**
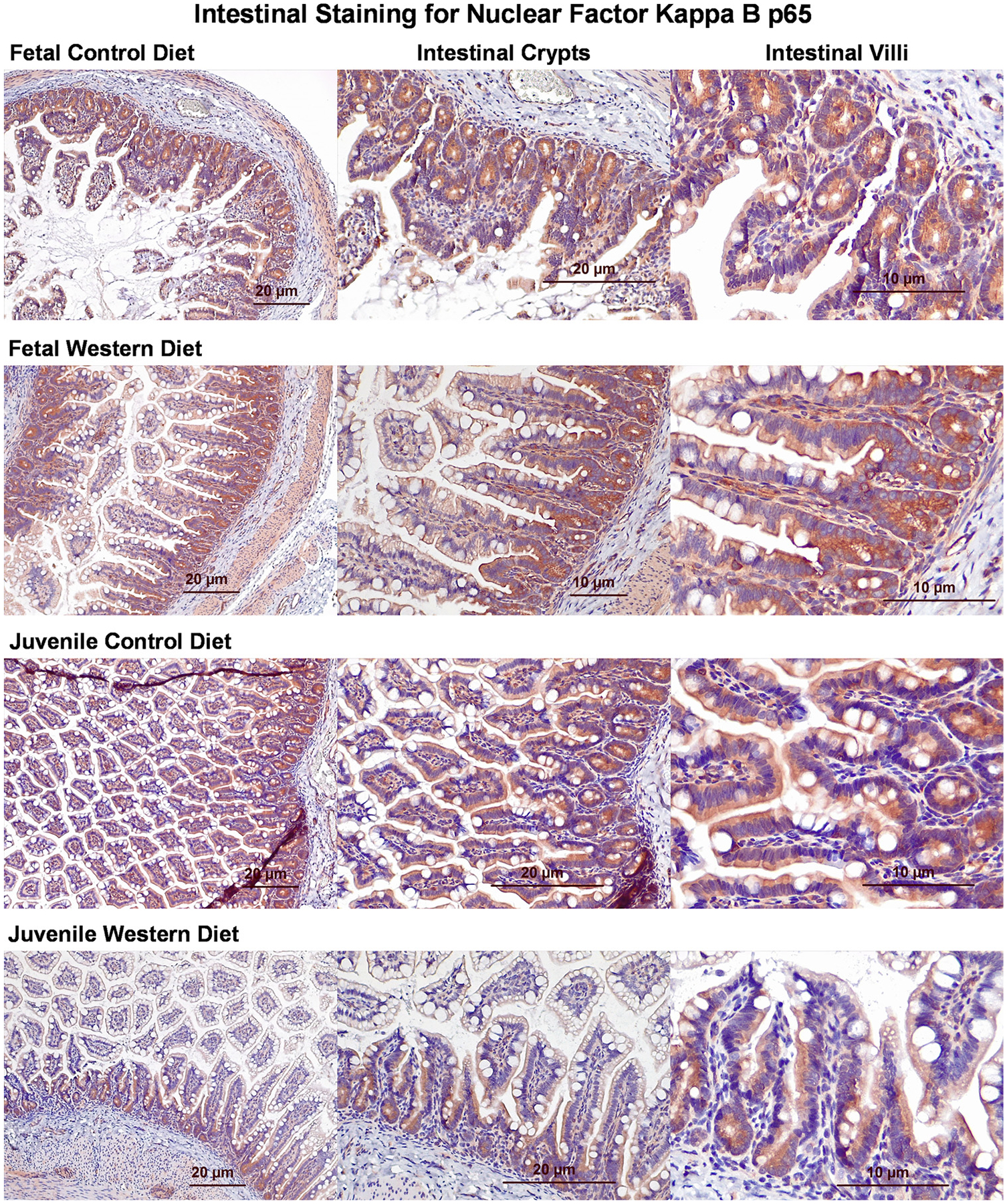
Immunohistochemical staining of histological slides for NF-κB p65. Microscopic examination of tissue obtained from a control diet (CD) and Western diet (WD) fetus and juveniles for NF-κB p65. NF-κB p65 appears red, whereas nuclear components appear blue.

On IHC staining, we found similar intensities of staining for both CD and WD fetuses and juveniles for p65 (Fig. 6).

### Longer Dam Exposure Led to Increased Inflammatory Milieu

We compared the levels of inflammatory markers in the fetal and juvenile intestines of dams who had been maintained on WD diet for longer periods of time. This includes fetal tissue from dams on the diet for 3 mo (F3M) and 1 year (F1Y) and juvenile tissue from dams on the diet for 2 year (J2Y) and 3 years (J3Y).

**Figure 7.**
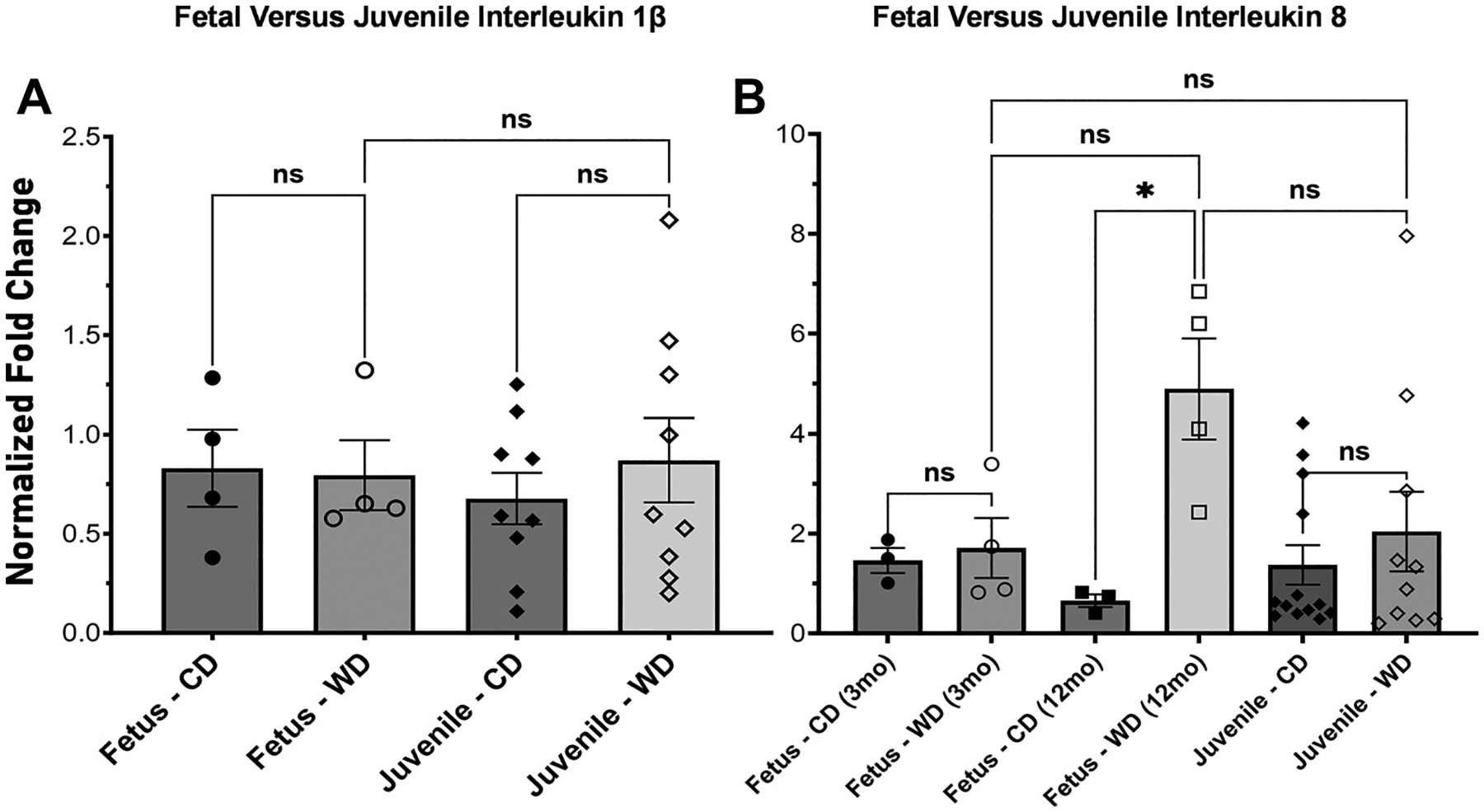
Expression of interleukin *1β* and *8* across generations via RT-qPCR. Expression of *IL-1β* (*A*) and *IL-8* (*B*) between fetal tissue [3 mo on diet (F3M) or 1 yr on diet (F1Y)] and juvenile tissue [2 yr on diet (J2Y)]. For *IL-1β*, we found there was no significant change from year to year (*P* = 0.994). There was no significant change in *IL-8* from year to year (F3M to F1Y *P* = 0.1496, F1Y to J2Y *P* = 0.1046, F3M to J2Y *P* = 0.9996). Error bars represent means ± SE. ns, not significant. * indicates significance.

Levels of fetal baboon ileum *IL-1β* mRNA were similar in F3M and J3Y (*P* = 0.99996; Fig. 7A). Ileum *IL-8* mRNA from WD3M fetuses had similar expression (*P* > 0.9999). F1Y ileum *IL-8* mRNA had significantly elevated levels (*P* = 0.0426). J3Y *IL-8* mRNA was not statistically significant (*P* = 0.9457). There was no significant difference between F3M, F1Y, and J2Y (Fig. 7B).

**Figure 8.**
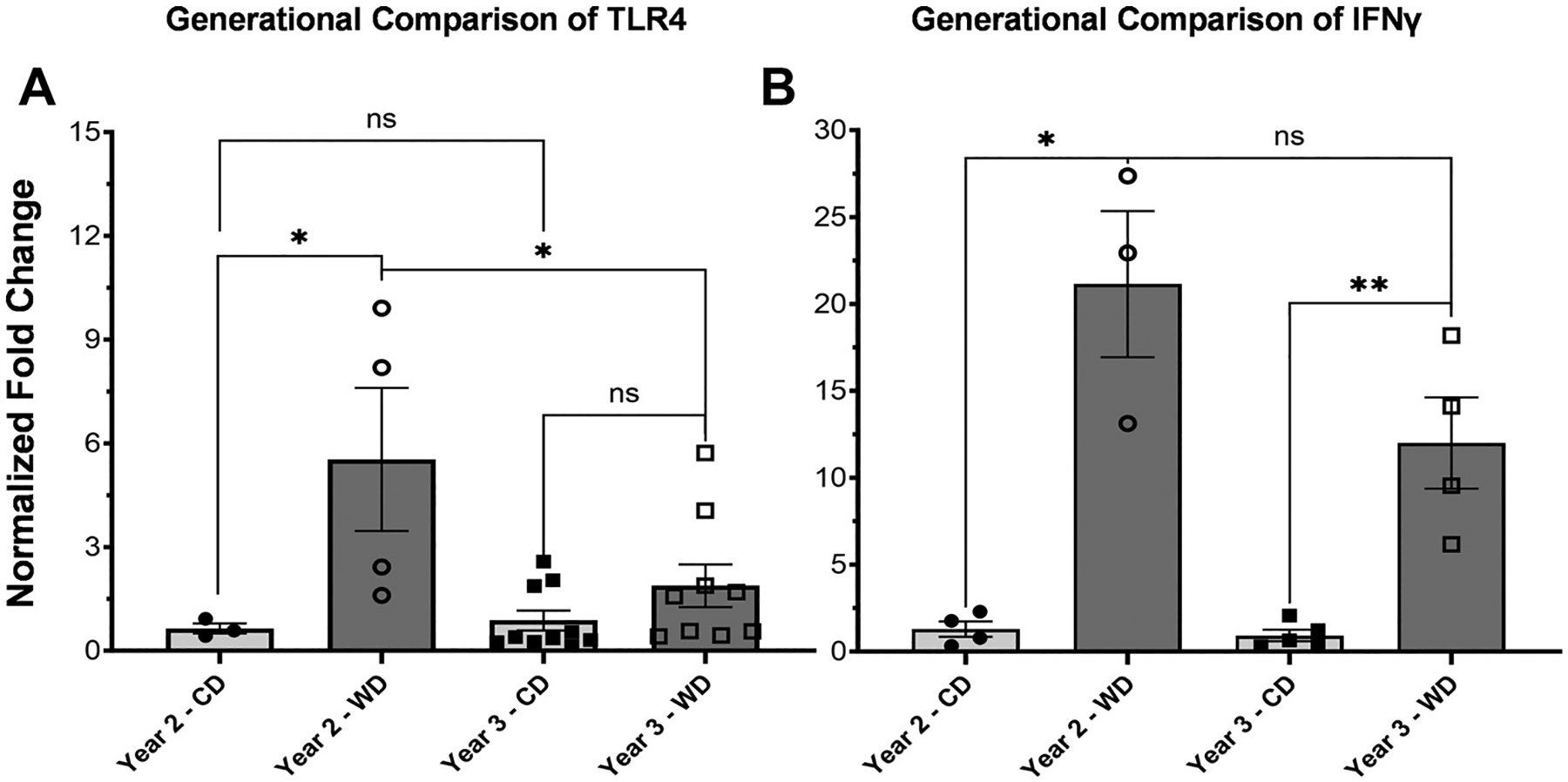
Expression of Toll-like receptor 4 and interferon gamma (*IFNγ*) across generations via RTqPCR. *A*: expression of *TLR4* between juvenile tissue. We found that *TLR4* expression was significantly elevated in juvenile tissue 2 yr on diet (J2Y) (*P* = 0.0186). In J3Y, there was a trend toward higher levels but this difference was not statistically significant (*P* = 0.6914). There was a significant decrease between J2Y and J3Y from WD dams (*P* = 0.02687). *B: IFNγ* in J2Y was significantly elevated (*P* = 0.0242). In J3Y, there was also a significant elevation (*P* = 0.0021). There was no significant difference between J2Y and J3Y (*P* = 0.7991). Error bars represent means ± SE. ns, not significant. * indicates significance near *P* < 0.05. ** indicates signficance at *P* < 0.005.

Ileum *TLR4* mRNA was significantly elevated in WD J2Y (*P* = 0.0186). In J3Y, there were no differences in *TLR4* mRNA (*P* = 0.6914). There was a significant decrease in *TLR4* between J2Y and J3Y from WD dams (*P* = 0.02687; Fig. 8A).

Ileum *IFNγ* mRNA J2Y was significantly elevated (*P* = 0.0242). In J3Y, ileum *IFNγ* mRNA was also significantly elevated (*P* = 0.0021). There was no significant difference between years (*P* = 0.7991; Fig. 8B).

### NEC Induction Results in Elevated Inflammatory Markers in Western Diet Enteroids

**Figure 9.**
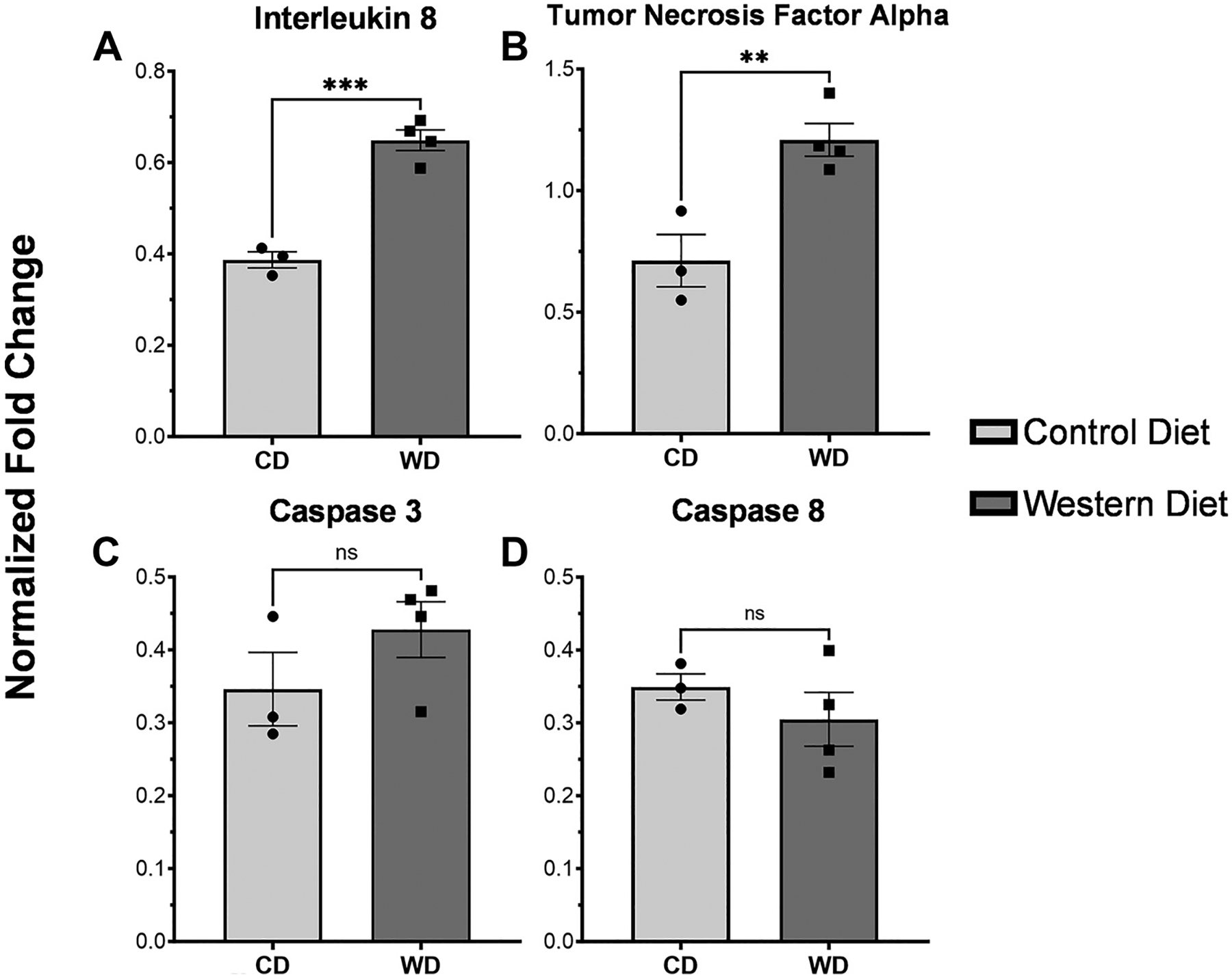
Expression of inflammatory and apoptotic markers between control diet and Western diet baboon enteroids. Western diet enteroids exposed to necrotizing enterocolitis (NEC) conditions showed a significant elevation in inflammatory markers when compared with control diet enteroids. This includes *IL-8* (*P* = 0.0004) (*A*) and *TNFα* (0.0092) (*B*). There was no statistically significant difference between either *caspase 3 (C)* or *caspase 8* (*D*) (*P* = 0.2436 and *P* = 0.3799, respectively). ns, not significant. ** indicates significance near *P* < 0.005. *** indicates significance near *P* < 0.0005.

Enteroids derived from fetuses from WD dams exposed to NEC conditions showed a significant elevation in inflammatory markers when compared with CD enteroids. This includes *IL-8* (*P* = 0.0004) and *TNFα* (0.0092). When we examined markers of apoptosis, there was no statistically significant difference between either *caspase 3 or 8* (*P* = 0.2436 and 0.3799) (Fig. 9).

### Barrier Effects

**Figure 10.**
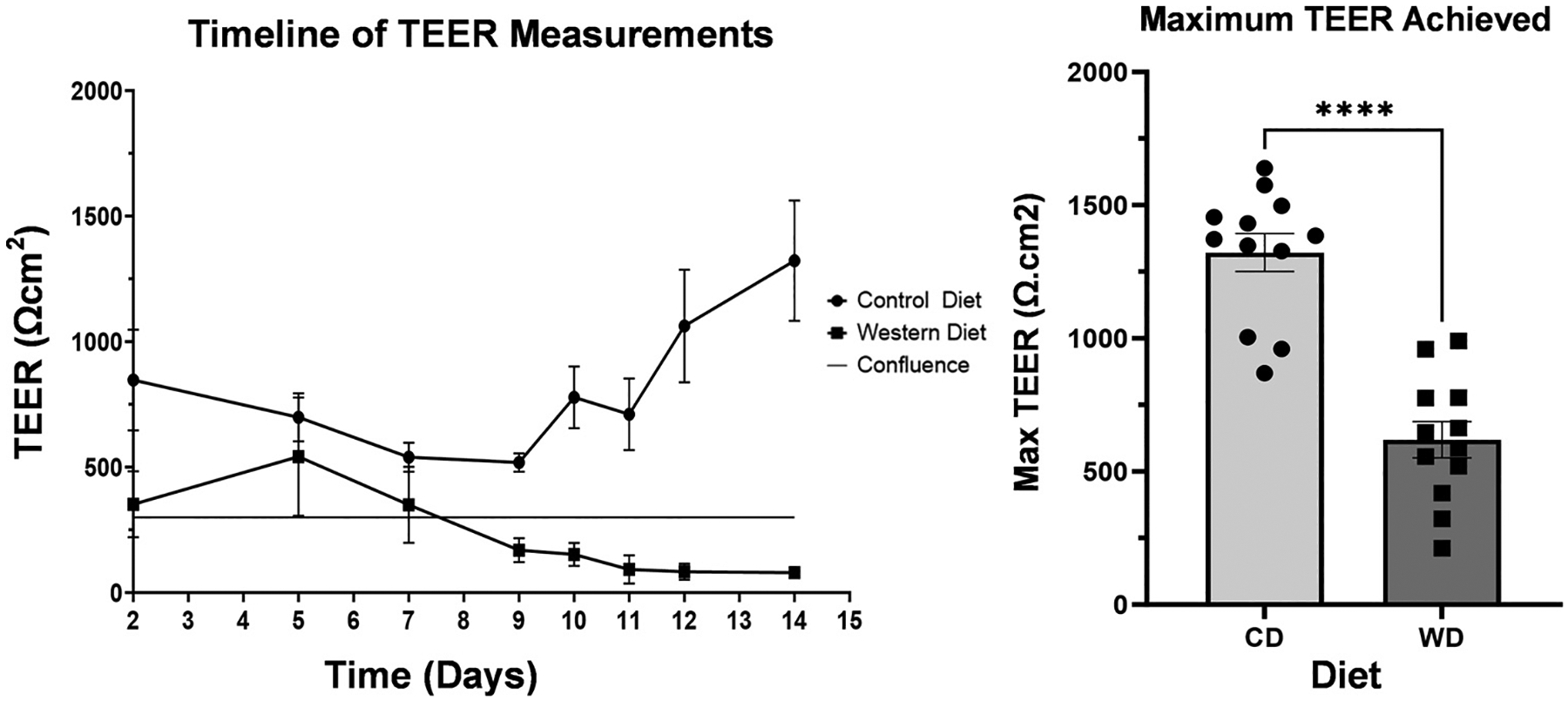
Barrier function of baboon control diet vs. Western diet enteroids through transepithelial electrical resistance (TEER) measurement. The maximum TEER achieved was 1638 Ωcm^2^ for the control diet with an average maximum of 1488.2 Ω cm^2^. The maximum for the Western diet was 959 Ω cm^2^ with an average maximum of 467.3 Ω cm^2^. When comparing maximums, there is a statistically significant difference between the control diet and Western diet enteroids (*P* < 0.0001). **** indicates significance near *P* < 0.0001.

Our experiment lasted 36 days. This experiment was done with a different pair of baboon fetus enteroids. The maximum TEER achieved was 1638 Ω/cm^2^ for the CD with an average maximum of 1488.2 Ω/cm^2^. The maximum for the WD was 959 Ω/cm^2^ with an average maximum of 467.3 Ω/cm^2^. When comparing maximums, there is a statistically significant difference between the CD and WD (*P* < 0.0001). On visual microscopic inspection, the WD enteroids achieved confluence and rapidly declined after differentiation. CD enteroids also achieved confluence and rapidly increased in TEER after differentiation (Fig. 10).

**Figure 11.**
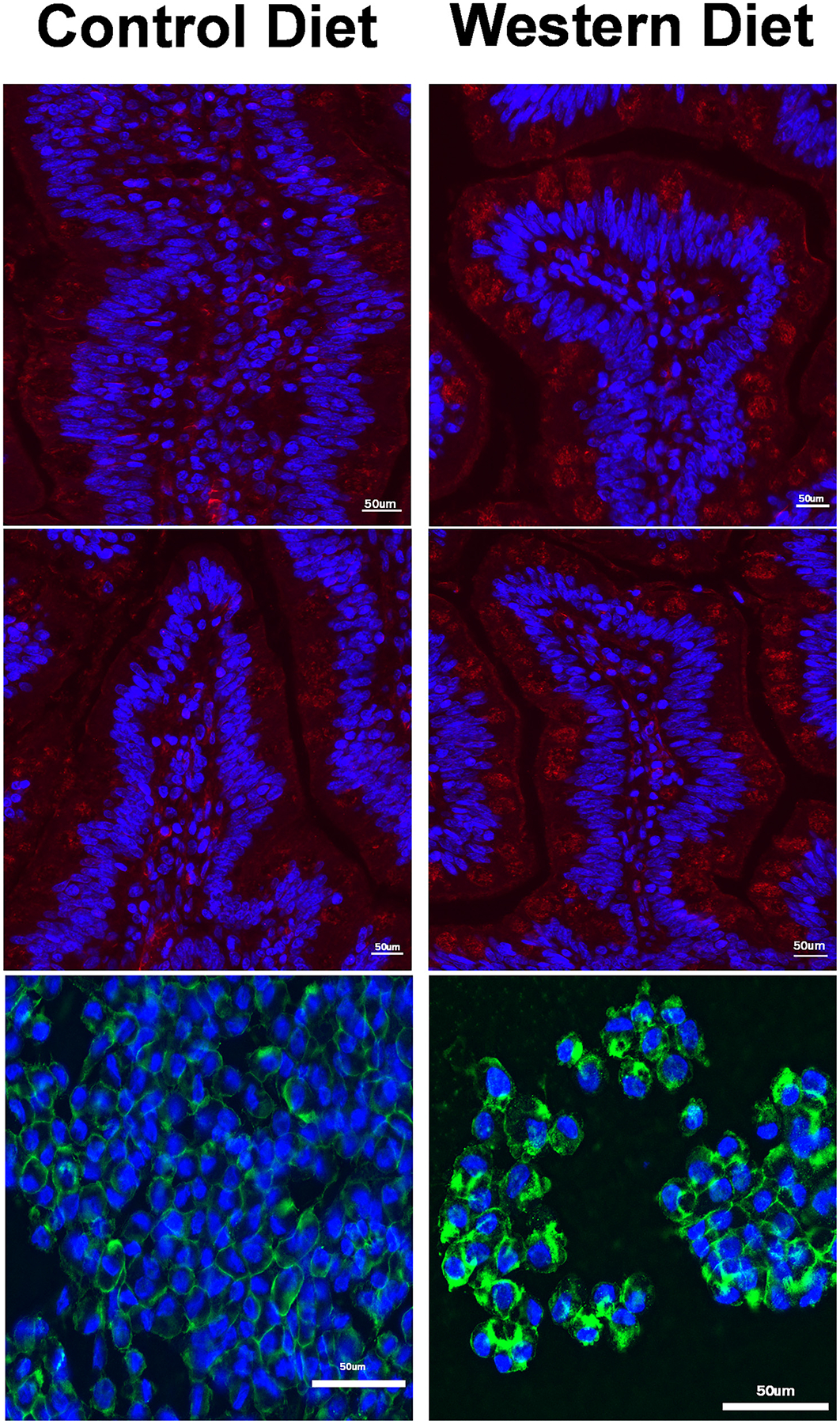
Immunofluorescent staining of histological slides for apoptosis and TransWell cells for claudin-1. Microscopic examination of cells stained using the ApoTag Red In Situ Apoptosis Detection Kit is shown in red and blue. Claudin-1 staining (in blue and green) of control and Western diet enteroids is shown as well. No statistical analysis could be performed for either staining.

Fluoroscopic examination of TEER membranes showed that the CD had organized and appropriately positioned claudin-1. When we examined the WD, the positioning of claudin-1 was much more disorganized and more cytoplasmic instead of at the plasma membrane (Fig. 11). ApoTag staining also showed no major difference in markers of apoptosis (Fig. 11).

**Figure 12.**
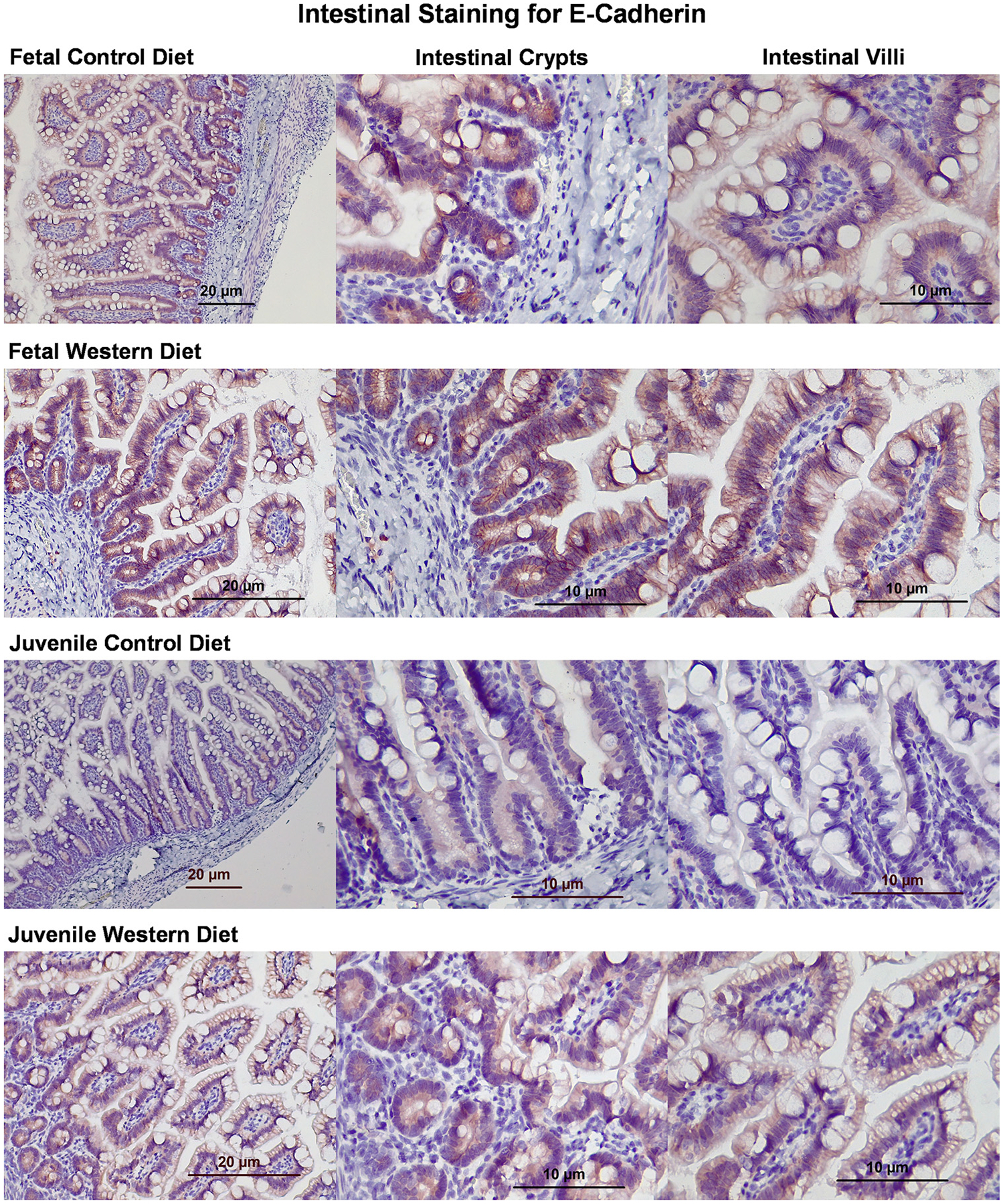
Immunohistochemical staining of histological slides for E-cadherin. Microscopic examination of tissue obtained from a control diet (CD) and Western diet (WD) fetus and juvenile for E-cadherin. E-cadherin appears red, whereas nuclear components appear blue.

**Figure 13.**
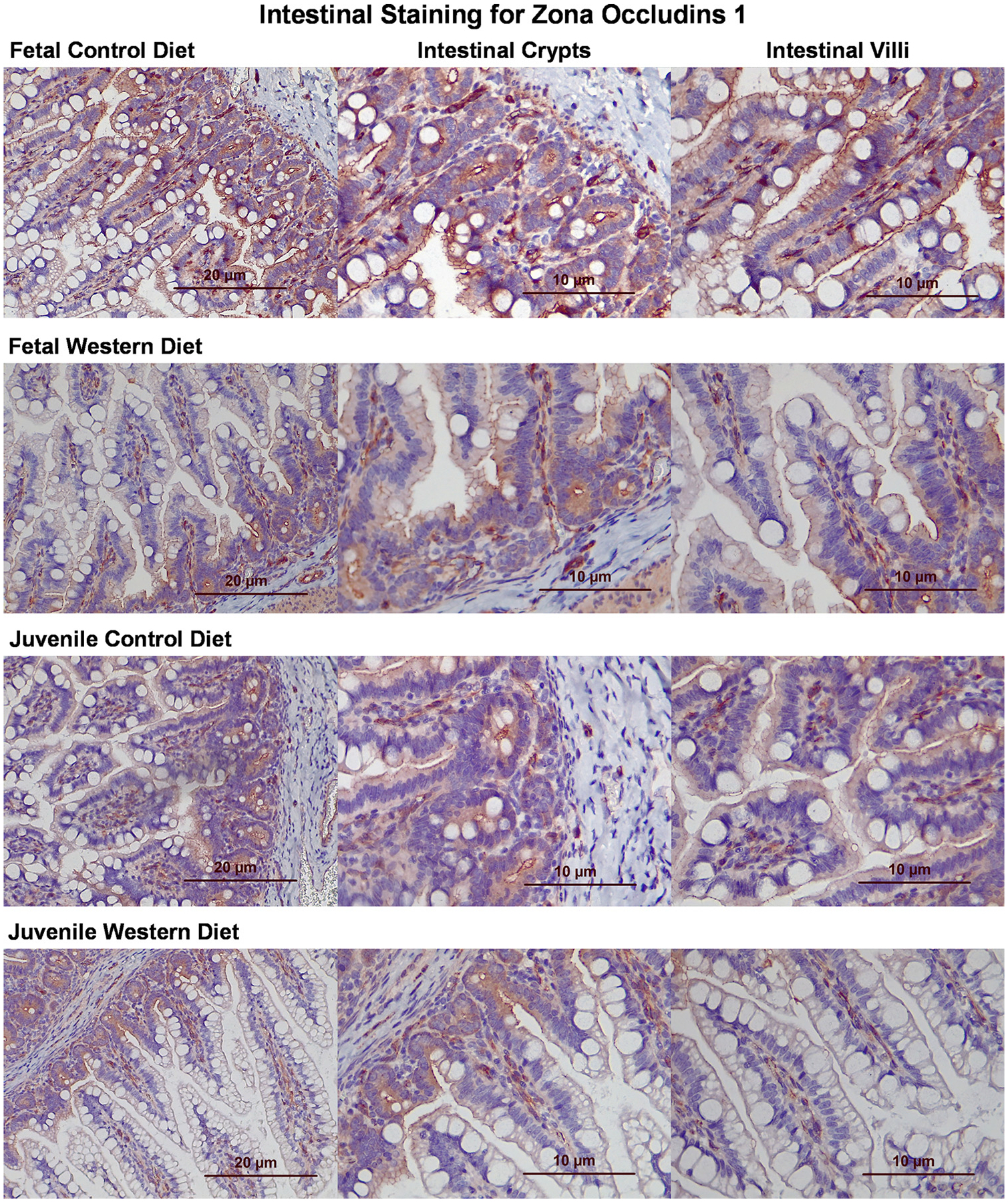
Immunohistochemical staining of histological slides for zona occludins 1 (ZO-1). Microscopic examination of tissue obtained from a control diet (CD) and Western diet (WD) fetus and juveniles for ZO-1. ZO-1 appears red, whereas nuclear components appear blue.

IHC staining of fetal tissue for the barrier protein E-cadherin showed similar levels of expression, with a possible slightly stronger staining in cells of WD fetuses. Juvenile tissue had noticeably more intense staining of E-cadherin in WD tissue compared with CD tissue, especially in intestinal villi (Fig. 12). In fetal intestinal tissue, staining for ZO-1 showed stronger staining in the CD compared with WD samples. This was especially true in the intestinal villi. In the juvenile samples, there were similar levels of ZO-1 in the intestinal crypts. There is a slightly stronger staining of the villi of CD juveniles. Villi of WD juveniles appear to have a higher amount of fat globules compared with WD villi (Fig. 13).

### Proliferating Cell Nuclear Antigen Levels, but not *K*_i_-67 Levels, are Elevated in Western Diet Fetuses

**Figure 14.**
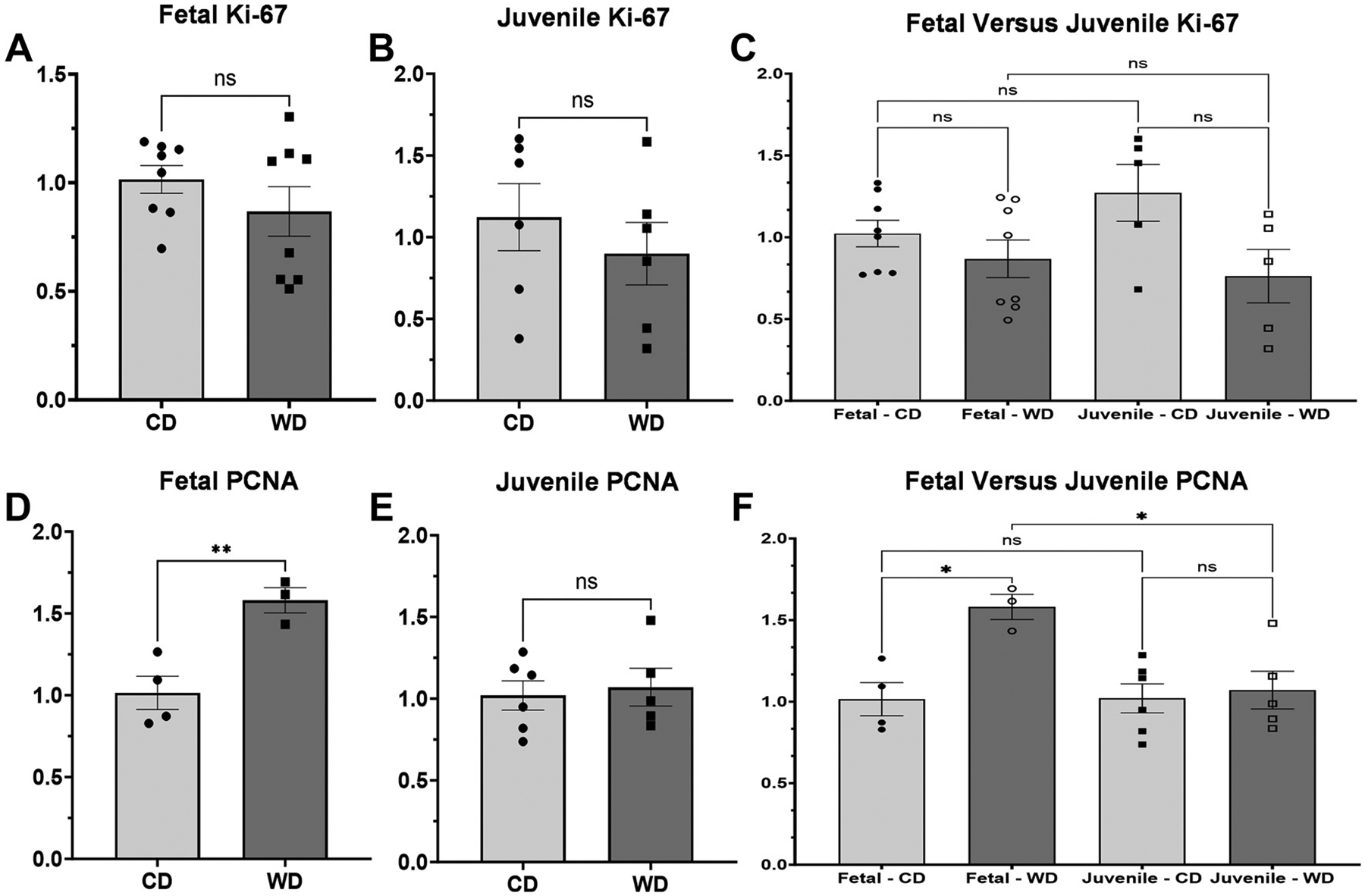
Markers of proliferation in fetal and juvenile tissue. *A*–*C*: fetal and juvenile tissue showed similar levels of *K*_*i*_-*67* between control diet (CD) and Western diet (WD) (*P* = 0.279 and *P* = 0.444, respectively). There were no significant differences in any comparison between fetuses and juveniles (fetal CD vs. juvenile CD *P* = 0.533; fetal WD vs. juvenile WD *P* = 0.937). *D*–*F*: there were significantly elevated levels of proliferating cell nuclear antigen (*PCNA*) in fetal WD samples compared with controls (*P* = 0.009), but not in juveniles (*P* = 0.734). Fetal WD levels were significantly higher than juvenile WD levels (*P* = 0.029). There was no significant difference between fetal and juvenile CD samples (*P* > 0.999). ns, not significant. * indicates significance near *P* < 0.05. ** indicates significance near *P* < 0.005.

To analyze the role of proliferation in the maintenance of barrier function, we evaluated markers of proliferation. Proliferating cell nuclear antigen (*PCNA*) was significantly elevated in WD fetuses (*P* = 0.009) but not in juveniles (*P* = 0.734). We also assessed levels of *K*_*i-*_*67*, which showed no significant difference between WD and CD levels for fetuses or juveniles (*P* = 0.279 and *P* = 0.444, respectively). There were significant differences between fetal WD and juvenile WD levels (*P* = 0.029), but all other comparisons were not significantly different (fetal CD vs. juvenile CD *P* > 0.9999; Fig. 14).

## DISCUSSION

Obesity and the Western diet play an established role in maternal and fetal pathophysiology (6), with effects ranging from lower APGAR scores to increased risk of metabolic syndrome. Although the maternal baboons had not met the criteria for obesity, they were all sustained on a WD, which allows us to evaluate the difference between maternal diet alone versus the effects of obesity. Offspring from dams fed a WD had increased levels of select inflammatory markers in their intestinal tissue. This trend was persistent across generations and showed an increasing trend the longer dams were exposed to this diet. The implications of this intestinal inflammatory state are seen when we examine barrier function, in which we found decreased function. These findings provide insight into a possible etiology of fetal gastrointestinal complications from maternal exposure to the WD, suggesting a potential therapeutic target for soon-to-be mothers.

In dams, as mentioned, the WD was not associated with statistically different weight gain or change in waist circumference. However, there was a statistically different change in SSF. This could signify changes in fat deposition or increased fat accumulation. We previously reported that dams fed the WD at 0.6 gestation exhibited significantly elevated plasma CRP but not IL-6 (22). In addition, dams had elevated circulating neutrophil numbers consistent with mild systemic inflammation. The WD-fed dams also exhibited a greater glucose area under the curve response in the intravenous glucose tolerance test (IVGTT), and at the time of IVGTT, increased fasting blood glucose. These dams also exhibited significantly elevated plasma triglycerides and cholesterol levels.

Several markers of intestinal inflammation were increased in our study. A study by Bekkering et al. (26) showed that exposure of the innate immune system to sterile danger signals such as lipid metabolites, abundant glucose, fatty acids (FA), or glycation end products could lead to a maladaptive trained immunity. This maligned immune system leads to sterile inflammation, leading to increased IL-1β production and migration of immune cells into adipose stores (27). This is associated with the TLR4/NLR family pyrin domain containing 3 (NLRP3) pathway (28). TLR4 and its impact on inflammation and obesity have been previously studied (29–31), and some have examined the impact of WD on TLR4. A recent study by Malesza et al. (32) showed that TLR4 was directly stimulated by saturated fatty acids, resulting in NF-κB pathway activation and increased IL-6 and TNFα synthesis. When we examined the various markers of NF-κB pathway activation, however, there was no significant difference between the diets. A paper by Liu et al. (33) shows that NF-κB activation and signaling led to multiple pathway activations. For inflammation, IL-12 was released. IL-18 release led to angiogenesis, and IL-23 and IL-12 led to T-cell activation, leading to differentiation into inflammatory T-cells and memory T-cells.

Previous studies have shown that NF-κB is a primary regulator of the inflammatory response in obesity (34, 35), but few studies have examined the direct effect of the WD on this pathway. One study found that a high-fat diet impaired liver regeneration. This was due to increased production of the NF-κB inhibitor I-kappa-B-alpha (IκBα) (36). Another possible explanation is that the inflammation seen is not NF-κB-related, but polyunsaturated fatty acids (PUFAs) induced. These are fatty acids (FAs) that contain more than one carbon double bond. The main long-chain PUFAs are the omega-3 (n-3) PUFAs and the omega-6 (n-6) PUFAs. The Western diet has been shown to have increased levels of n6 PUFAs. The average n6/n3 ratio of food was 1:1, but most recently has been as high as 16:1 (37). This is a potential future area of study.

In contrast intestinal IL-10, although not significantly different, had a trend toward elevation. This is typically an anti-inflammatory marker known to inhibit the production/synthesis of all of the abovementioned markers (38). Although the precise mechanisms underlying these changes are unknown, they may, at least in part, be associated with differences in the cellular response to the inflammatory milieu. Although most studies have examined IL-10 levels in obesity, Hong et al. (39) found that mice who overexpressed IL-10 were more sensitive to insulin and had skeletal muscle protection from macrophage infiltration after exposure to a high-fat, high-sugar (HFHS) diet. Cholesterol levels have also been found to inversely correlate with serum IL-10 levels (40). In studies of obesity, levels of IL-10 were found to be elevated in individuals with obesity (40, 41). They have also found that in patients with obesity, low levels of IL-10 were associated with type II diabetes and metabolic syndrome (40–42).

Although these markers are typically associated with the acute phase of inflammation, many also play a significant impact in chronic inflammation. A study by Dinarello and Van der Meer (43) illustrated that patients with chronic conditions such as gout, congestive heart failure, and osteoarthritis had improvement in their disease states after being treated with an IL-1 inhibitor (44). TLR4 has been implicated in several similar diseases, particularly inflammatory bowel disease (45). IFNγ being significantly elevated also points toward long-term exposure to an inflammatory environment. IFNγ has been implicated in chronic inflammatory diseases such as psoriasis (46). The implications of this low-grade chronic inflammatory state were also examined in our study. Previous studies have shown that patients with NEC have a proclivity to the hyperinflammatory state (47). Other studies have also shown that higher levels of IL-8 (48, 49) and TNFα (50, 51) coincide with increased severity of NEC. After NEC induction, our enteroids from the WD group had a stronger response to the stimulus with significantly elevated levels of both IL8 and TNFα. This could indicate that exposure to the WD and its low-grade inflammatory state prime the neonate bowel for an inflammatory response.

Inflammation has similarly been implicated as part of intestinal barrier failure, which is another facet of NEC pathogenesis (52). The cascade of the “leaky gut” starts with inflammatory markers released in response to LPS or other endotoxins (53). By TEER measurement, enteroids from fetuses of WD dams were noted to have decreased barrier function at baseline. The WD has been shown to alter barrier function in rats, leading to metabolic endotoxemia (54). In mice exposed to similar conditions as our baboons, the WD was associated with decreased defensin expression. High fructose uptake was also associated with increased endotoxin translocation (55).

Cadherins are a family of transmembrane proteins. The main function of these proteins is in cell-to-cell adhesion in a Ca^2+^-dependent manner (56). E-cadherin is a part of this family and is considered the prototypical member. Its main function is the formation of adherens junctions (AJs) by binding catenins (such as β-catenin; 57). When we performed IHC for this protein, we found stronger staining in WD fetuses and noticeably stronger staining in WD juveniles (especially in the villi). One possible explanation is the internalization of E-cadherin, which is the process of being endocytosed from the membrane. Although this is currently an area of active study, there have been physiological triggers for E-cadherin internalization, such as cell movement or cell polarity, and several pathological triggers (58, 59). Studies have illustrated internalization due to exposure to inflammatory cytokines (60–62). Other studied triggers include calcium depletion and exposure to oxygen radicals (63, 64). In human neonates, this internalization is also seen in the pathology of necrotizing enterocolitis (NEC) and is similarly seen in patients with inflammatory bowel disease (IBD) such as Crohn’s disease (CD) or ulcerative colitis (UC; 65, 66).

Internalization of TJ proteins may also explain the disorganized localization of claudin-1. Claudins are membrane proteins that primarily maintain barrier function. Unlike the AJ of E-cadherin, claudins tend to form tight junctions (TJs) (67). Like E-cadherin, claudin-1 has been found to be endocytosed during times of inflammation. A study by Bruewer et al. incubated cells with IFNγ, and found that although claudin-1 levels stayed the same, there was a significant redistribution toward internalization. This was similar for other members of the claudin family and ZO-1 (68). ZO-1 showed stronger staining in fetal CD tissue over WD tissue. In inflammatory states, ZO-1 has been found to have decreased mRNA and protein expression in the intestine (69). Similar to E-cadherin, ZO-1 was found to be significantly decreased in NEC as well (70).

We additionally saw higher levels of fat globules in the fetal intestine from the WD (most in the ZO-1 staining). These increased fat globules can lead to several problems with barrier integrity. One is that they increase local pressure on enterocytes. This increased pressure can cause loosening of TJs, allowing for increased permeability (71, 72). In addition, an increased fat content (specifically chylomicrons) can allow for increased absorption of LPS (73). LPS is a key bacterial endotoxin and one of the most potent inducers of inflammation via the TLR4 receptor (74). It has been implicated in several intestinal pathologies, including NEC and the inflammatory bowel disease (75, 76).

We found that WD fetuses had elevated levels of PCNA. PCNA has several functions ranging from DNA synthesis to chromatin assembly and even RNA transcription (77). It is also important in DNA repair. It does this by binding to the DNA polymerase δ and ɛ (78). A study by Conlon et al. (79) shows that rats fed a Western-style diet had increased colonocyte DNA single-strand breaks, indicating higher levels of DNA damage. Thus, the elevated levels of PCNA seen in our study likely correlate to higher amounts of DNA damage in fetuses and higher amounts of enterocyte proliferation in the setting of fetal bowel development (80). This is bolstered by the lack of significance in the juvenile groups. Once off the WD, levels of PCNA returned to their baseline.

We did not find any significant difference in the levels of *K*_i_-67. Commonly seen in cancer research, this protein typically functions as a marker of cell proliferation in tumors. It works as a surfactant, preventing chromosomes from self-binding after the nuclear envelope has broken down. It also facilitates the binding of the mitotic spindle to chromosomes (81). Unlike PCNA, it is not known for its function in DNA repair. In the study by Doganay et al., rats were exposed to a CD, WD, and a ketogenic diet (KD). Levels of *K*_i_-67 in the liver increased the least in the WD group and the most in the KD group (82). Our findings are also congruent with the previous literature. Kullmann et al. (83) showed that in biopsies of inflammatory bowel disease for dysplasia, PCNA levels were significantly higher than *K*_i_-67.

Although we had an adequate sample size to investigate our hypothesis, an increase in sample size would improve statistical power and further delineate the significance between the two diets. Our study would also benefit from examining the protein landscape of these samples via Western blot, ELISA, or an unbiased proteomics approach. Similarly, ribonucleic acid sequencing (RNA-Seq) could be applied for an unbiased approach to evaluating global gene expression patterns in the intestinal epithelium. This could elucidate whether the gene expression correlates with cellular function and protein synthesis at the time of harvest. In addition, other pathways of inflammation are worth exploring. Our study limited its focus to common markers associated with inflammation. Pathways such as aryl hydrocarbon receptor (AHR) (84), A20 (85), resistin (86), and others warrant exploration, given their roles in inflammation. Finally, our laboratory has examined the possible effects of polyunsaturated fatty acids (PUFAs; 87), possibly leading to ferroptosis in NEC. Further experimentation into how maternal diet plays into this pathway is needed.

## Figures and Tables

**Table 1.** List of genes and primer sequences

Gene	Forward	Reverse
*IL-1β*	5′-TGTACGATCACTGAACTGCA-3′	5′-GAAGTCAGTTATATCCTGGC-3′
*IL-8*	5′-CCTTTCCACCCCAAATTTATC-3′	5′-TTCTGTATTGACGCAGTGTGG-3′
*IL-10*	5′-AAACCACAAGACAGACTT-3′	5′-GATTTTGGAGACCTCTAATTTA-3′
*IL-12*	5′-CGTGCCTTCACCACTCCCAA-3′	5′-CAGGCCTCTACTGTGCTGGTT-3′
*IL-18*	5′-TAGCTGAAGATGATGAAAACCTGGA-3′	5′-TAGGGGCCGATTTCCTTGGT-3′
*IL-23*	5′-CGCCCCTTCTACAGCCATTTC-3′	5′-TGAGTCGTGTGGTTGCTGTGA-3′
*IFNγ*	5′-GCAGTTGATCCAGATGTAGCAG-3′	5′-CCTTGATGGTCTCCACACTCTT-3′
*TNFα*	5′-TCTTCACTGGAAAGGACACCA-3′	5′-GAAGGAGAAGAGGCTGAGGAA-3 ‘
*TLR4*	5′-TCATTTTCCCTGGTGAGTGTGAA-3′	5′-GTTGGCAGTGAAAGTAAGCCT-3′
*Caspase 3*	5′-CTGCTCTCGGGCGGTTGTG-3′	5′-TTCCAGAGTCCACTGATTTGCTTCC-3′
*Caspase 8*	5′-ATTCAGCAGAGGGAGAACAAGG-3′	5′-GACAGTATCCCCGAGGTTTGC-3′
*Ki-67*	5′-AATCGGACACTACCGCTGGAC-3′	5′-CACCAAAGGACACACGCCTTC-3′
*PCNA*	5′-CTGGCTATGGGCGTGAACCT-3′	5′-AGCGCCAAGGTATCCGCATTA-3′
*GAPDH*	5′-CCAAAATCAAGTGGGGCGAT-3′	5′-GGTCATGAGTCCTTCCACGA-3′

A comprehensive list of all genes examined via PCR as well as their respective forward and reverse primers. PCNA, proliferating cell nuclear antigen; TLR4, Toll-like receptor 4.

**Table 2.** Lack of significant differences between sexes

Gene	*P* Value
*Fetal*	
*IL-1β*	0.7055
*IL-8*	0.7820
*Juvenile*	
*IL-1β*	0.3825
*IL-6*	0.4093
*IL-10*	0.8596
*IFNγ*	0.2947

When similar genes were evaluated for significance based on the sex of the fetus or juvenile, none were found. The table shows these respective *P* values.

## Data Availability

Data will be made available upon reasonable request.

## References

[1] Vucenik I, Stains JP. Obesity and cancer risk: evidence, mechanisms, and recommendations. Ann N Y Acad Sci 1271: 37–43, 2012. doi:10.1111/j.1749-6632.2012.06750.x. 23050962 PMC3476838

[2] Ri M, Aikou S, Seto Y. Obesity as a surgical risk factor. Ann Gastroenterol Surg 2: 13–21, 2018. doi:10.1002/ags3.12049. 29863119 PMC5881295

[3] Rana JS, Nieuwdorp M, Jukema JW, Kastelein JJP. Cardiovascular metabolic syndrome – an interplay of, obesity, inflammation, diabetes and coronary heart disease. Diabetes Obes Metab 9: 218–232, 2007. doi:10.1111/j.1463-1326.2006.00594.x. 17391148

[4] Verma S, Hussain ME. Obesity and diabetes: an update. Diabetes Metab Syndr 11: 73–79, 2017. doi:10.1016/j.dsx.2016.06.017. 27353549

[5] Leddy MA, Power ML, Schulkin J. The impact of maternal obesity on maternal and fetal health. Rev Obstet Gynecol 1: 170–178, 2008. 19173021 PMC2621047

[6] Schmatz M, Madan J, Marino T, Davis J. Maternal obesity: the interplay between inflammation, mother and fetus. J Perinatol 30: 441–446, 2010. doi:10.1038/jp.2009.182. 19907427

[7] Catalano PM. Obesity and pregnancy—the propagation of a viscous cycle? J Clin Endocrinol Metab 88: 3505–3506, 2003. doi:10.1210/jc.2003-031046. 12915626

[8] Radaelli T, Uvena-Celebrezze J, Minium J, Huston-Presley L, Catalano P, Hauguel-de Mouzon S. Maternal interleukin-6: marker of fetal growth and adiposity. J Soc Gynecol Investig 13: 53–57, 2006. doi:10.1016/j.jsgi.2005.10.003. 16378913

[9] Sindhu S, Thomas R, Shihab P, Sriraman D, Behbehani K, Ahmad R. Obesity is a positive modulator of IL-6R and IL-6 expression in the subcutaneous adipose tissue: significance for metabolic inflammation. PLoS One 10: e0133494, 2015. doi:10.1371/journal.pone.0133494. 26200663 PMC4511728

[10] Paepegaey A-C, Genser L, Bouillot J-L, Oppert J-M, Clément K, Poitou C. High levels of CRP in morbid obesity: the central role of adipose tissue and lessons for clinical practice before and after bariatric surgery. Surg Obes Relat Dis 11: 148–154, 2015. doi:10.1016/j.soard.2014.06.010. 25393045

[11] Hurlimann J, Thorbecke GJ, Hochwald GM. The liver as the site of c-reactive protein formation. J Exp Med 123: 365–378, 1966. doi:10.1084/jem.123.2.365. 4379352 PMC2138142

[12] Camargo A, Delgado-Lista J, Garcia-Rios A, Cruz-Teno C, Yubero-Serrano EM, Perez-Martinez P, Gutierrez-Mariscal FM, Lora-Aguilar P, Rodriguez-Cantalejo F, Fuentes-Jimenez F, Tinahones FJ, Malagon MM, Perez-Jimenez F, Lopez-Miranda J. Expression of proinflammatory, proatherogenic genes is reduced by the Mediterranean diet in elderly people. Br J Nutr 108: 500–508, 2012. doi:10.1017/S0007114511005812. 22085595

[13] Devêvre EF, Renovato-Martins M, Clément K, Sautès-Fridman C, Cremer I, Poitou C. Profiling of the three circulating monocyte sub-populations in human obesity. J Immunol 194: 3917–3923, 2015. doi:10.4049/jimmunol.1402655. 25786686

[14] Kolb R, Sutterwala FS, Zhang W. Obesity and cancer: inflammation bridges the two. Curr Opin Pharmacol 29: 77–89, 2016. doi:10.1016/j.coph.2016.07.005. 27429211 PMC4992602

[15] Mozaffarian D Dietary and Policy priorities for cardiovascular disease, diabetes, and obesity. Circulation. 133: 187–225, 2016. doi:10.1161/CIRCULATIONAHA.115.018585. 26746178 PMC4814348

[16] Atkinson FS, Foster-Powell K, Brand-Miller JC. International tables of glycemic index and glycemic load values: 2008. Diabetes Care 31: 2281–2283, 2008. doi:10.2337/dc08-1239. 18835944 PMC2584181

[17] Tall AR, Yvan-Charvet L. Cholesterol, inflammation and innate immunity. Nat Rev Immunol 15: 104–116, 2015. doi:10.1038/nri3793. 25614320 PMC4669071

[18] Herzl E, Schmitt EE, Shearrer G, Keith JF. The effects of a Western diet vs. a high-fiber unprocessed diet on health outcomes in mice offspring. Nutrients. 15: 2858, 2023. doi:10.3390/nu15132858. 37447184 PMC10343591

[19] Huang C, Tan H, Song M, Liu K, Liu H, Wang J, Shi Y, Hou F, Zhou Q, Huang R, Shen B, Lin X, Qin X, Zhi F. Maternal Western diet mediates susceptibility of offspring to Crohn’s-like colitis by deoxycholate generation. Microbiome. 11: 96, 2023. doi:10.1186/s40168-023-01546-6. 37131223 PMC10155335

[20] Sikder MAA, Rashid RB, Ahmed T, Sebina I, Howard DR, Ullah MA, Rahman MM, Lynch JP, Curren B, Werder RB, Simpson J, Bissell A, Morrison M, Walpole C, Radford KJ, Kumar V, Woodruff TM, Ying TH, Ali A, Kaiko GE, Upham JW, Hoelzle RD, Cuív PÓ, Holt PG, Dennis PG, Phipps S. Maternal diet modulates the infant microbiome and intestinal Flt3L necessary for dendritic cell development and immunity to respiratory infection. Immunity 56: 1098–1114.e10, 2023. doi:10.1016/j.immuni.2023.03.002. 37003256

[21] Stumpf K, Sharma P, Brown LS, Brion LP, Mirpuri J. Maternal body mass index and necrotizing enterocolitis: a case-control study. PLoS One 19: e0296644, 2024. doi:10.1371/journal.pone.0296644. 38266000 PMC10807840

[22] Sugino KY, Mandala A, Janssen RC, Gurung S, Trammell M, Day MW, Brush RS, Papin JF, Dyer DW, Agbaga M-P, Friedman JE, Castillo-Castrejon M, Jonscher KR, Myers DA. Western diet-induced shifts in the maternal microbiome are associated with altered microRNA expression in baboon placenta and fetal liver. Front Clin Diabetes Healthc 3: 945768, 2022. doi:10.3389/fcdhc.2022.945768. 36935840 PMC10012127

[23] Buonpane C, Ares G, Yuan C, Schlegel C, Liebe H, Hunter CJ. Experimental modeling of necrotizing enterocolitis in human infant intestinal enteroids. J Invest Surg 35: 111–118, 2022. doi:10.1080/08941939.2020.1829755. 33100066 PMC8840553

[24] Li C, Jenkins S, Huber HF, Nathanielsz PW. Effect of maternal baboon (Papio sp.) dietary mismatch in pregnancy and lactation on post-natal offspring early life phenotype. J Med Primatol 48: 226–235, 2019. doi:10.1111/jmp.12415. 31025367 PMC6610582

[25] Maloyan A, Muralimanoharan S, Huffman S, Cox LA, Nathanielsz PW, Myatt L, Nijland MJ. Identification and comparative analyses of myocardial miRNAs involved in the fetal response to maternal obesity. Physiol Genomics 45: 889–900, 2013. doi:10.1152/physiolgenomics.00050.2013. 23922128 PMC3798778

[26] Bekkering S, Quintin J, Joosten LAB, van der Meer JWM, Netea MG, Riksen NP. Oxidized low-density lipoprotein induces long-term proinflammatory cytokine production and foam cell formation via epigenetic reprogramming of monocytes. Arterioscler Thromb Vasc Biol 34: 1731–1738, 2014. doi:10.1161/ATVBAHA.114.303887. 24903093

[27] Christ A, Lauterbach M, Latz E. Western diet and the immune system: an inflammatory connection. Immunity. 51: 794–811, 2019. doi:10.1016/j.immuni.2019.09.020. 31747581

[28] Nagareddy PR, Kraakman M, Masters SL, Stirzaker RA, Gorman DJ, Grant RW, Dragoljevic D, Hong ES, Abdel-Latif A, Smyth SS, Choi SH, Korner J, Bornfeldt KE, Fisher EA, Dixit VD, Tall AR, Goldberg IJ, Murphy AJ. Adipose tissue macrophages promote myelopoiesis and monocytosis in obesity. Cell Metab 19: 821–835, 2014. doi:10.1016/j.cmet.2014.03.029. 24807222 PMC4048939

[29] Fresno M, Alvarez R, Cuesta N. Toll-like receptors, inflammation, metabolism and obesity. Arch Physiol Biochem 117: 151–164, 2011. doi:10.3109/13813455.2011.562514. 21599616

[30] Könner AC, Brüning JC. Toll-like receptors: linking inflammation to metabolism. Trends Endocrinol Metab 22: 16–23, 2011. doi:10.1016/j.tem.2010.08.007. 20888253

[31] Velloso LA, Folli F, Saad MJ. TLR4 at the crossroads of nutrients, gut microbiota, and metabolic inflammation. Endocr Rev 36: 245–271, 2015. doi:10.1210/er.2014-1100. 25811237

[32] Malesza IJ, Malesza M, Walkowiak J, Mussin N, Walkowiak D, Aringazina R, Bartkowiak-Wieczorek J, Mądry E. High-fat, Western-style diet, systemic inflammation, and gut microbiota: a narrative review. Cells 10: 3164, 2021. doi:10.3390/cells10113164. 34831387 PMC8619527

[33] Liu T, Zhang L, Joo D, Sun S-C. NF-κB signaling in inflammation. Signal Transduct Target Ther 2: 17023–17023, 2017. doi:10.1038/sigtrans.2017.23. 29158945 PMC5661633

[34] Baker RG, Hayden MS, Ghosh S. NF-κB, inflammation, and metabolic disease. Cell Metab 13: 11–22, 2011. doi:10.1016/j.cmet.2010.12.008. 21195345 PMC3040418

[35] Carlsen H, Haugen F, Zadelaar S, Kleemann R, Kooistra T, Drevon CA, Blomhoff R. Diet-induced obesity increases NF-κB signaling in reporter mice. Genes Nutr 4: 215–222, 2009. doi:10.1007/s12263-009-0133-6. 19707810 PMC2745749

[36] DeAngelis RA, Markiewski MM, Taub R, Lambris JD. A high-fat diet impairs liver regeneration in C57BL/6 mice through overexpression of the NF-κB inhibitor, IκBα. Hepatology 42: 1148–1157, 2005. doi:10.1002/hep.20879. 16231352

[37] Simopoulos AP. The importance of the omega-6/omega-3 fatty acid ratio in cardiovascular disease and other chronic diseases. Exp Biol Med (Maywood) 233: 674–688, 2008. doi:10.3181/0711-MR-311. 18408140

[38] Lalani I, Bhol K, Ahmed AR. Interleukin-10: biology, role in inflammation and autoimmunity. Ann Allergy Asthma Immunol 79: 469–483, 1997 [Erratum in *Ann Allergy Asthma Immunol* 80: A-6, 1998]. doi:10.1016/S1081-1206(10)63052-9. 9433360

[39] Hong E-G, Ko HJ, Cho Y-R, Kim H-J, Ma Z, Yu TY, Friedline RH, Kurt-Jones E, Finberg R, Fischer MA, Granger EL, Norbury CC, Hauschka SD, Philbrick WM, Lee C-G, Elias JA, Kim JK. Interleukin-10 prevents diet-induced insulin resistance by attenuating macrophage and cytokine response in skeletal muscle. Diabetes 58: 2525–2535, 2009. doi:10.2337/db08-1261. 19690064 PMC2768157

[40] Kim SJ, Lim J, Nam GE, Park HS. Correlation between serum lipid parameters and interleukin-10 concentration in obese individuals. J Obes Metab Syndr 30: 173–177, 2021. doi:10.7570/jomes20122. 34011692 PMC8277584

[41] Esposito K, Pontillo A, Giugliano F, Giugliano G, Marfella R, Nicoletti G, Giugliano D. Association of low interleukin-10 levels with the metabolic syndrome in obese women. J Clin Endocrinol Metab 88: 1055–1058, 2003. doi:10.1210/jc.2002-021437. 12629085

[42] Blüher M, Fasshauer M, Tönjes A, Kratzsch J, Schön MR, Paschke R. Association of interleukin-6, C-reactive protein, interleukin-10 and adiponectin plasma concentrations with measures of obesity, insulin sensitivity and glucose metabolism. Exp Clin Endocrinol Diabetes 113: 534–537, 2005. doi:10.1055/s-2005-872851. 16235156

[43] Dinarello CA, van der Meer JWM. Treating inflammation by blocking interleukin-1 in humans. Semin Immunol 25: 469–484, 2013. doi:10.1016/j.smim.2013.10.008. 24275598 PMC3953875

[44] Ren K, Torres R. Role of interleukin-1β during pain and inflammation. Brain Res Rev 60: 57–64, 2009. doi:10.1016/j.brainresrev.2008.12.020. 19166877 PMC3076185

[45] Drexler SK, Foxwell BM. The role of Toll-like receptors in chronic inflammation. The Int J Biochem Cell Biol 42: 506–518, 2010. doi:10.1016/j.biocel.2009.10.009. 19837184

[46] Mehta NN, Teague HL, Swindell WR, Baumer Y, Ward NL, Xing X, Baugous B, Johnston A, Joshi AA, Silverman J, Barnes DH, Wolterink L, Nair RP, Stuart PE, Playford M, Voorhees JJ, Sarkar MK, Elder JT, Gallagher K, Ganesh SK, Gudjonsson JE. IFN-γ and TNF-α synergism may provide a link between psoriasis and inflammatory atherogenesis. Sci Rep 7: 13831, 2017. doi:10.1038/s41598-017-14365-1. 29062018 PMC5653789

[47] Snyder KB, Calkins CL, Golubkova A, Leiva T, Schlegel C, Hunter CJ. Despite recovery from necrotizing enterocolitis infants retain a hyperinflammatory response to injury. J Inflamm Res 17: 331–341, 2024. doi:10.2147/JIR.S436125. 38250141 PMC10800089

[48] Edelson MB, Bagwell CE, Rozycki HJ. Circulating pro- and counter-inflammatory cytokine levels and severity in necrotizing enterocolitis. Pediatrics. 103: 766–771, 1999. doi:10.1542/peds.103.4.766. 10103300

[49] Seo YM, Lin YK, Im SA, Sung IK, Youn YA; Department of Pediatrics, Seoul St. Mary’s Hospital, Catholic Medical College, Catholic University, Seoul, Korea. Interleukin 8 may predict surgical necrotizing enterocolitis in infants born less than 1500 g. Cytokine 137: 155343, 2021. doi:10.1016/j.cyto.2020.155343. 33128923

[50] Halpern MD, Clark JA, Saunders TA, Doelle SM, Hosseini DM, Stagner AM, Dvorak B. Reduction of experimental necrotizing enterocolitis with anti-TNF-α. Am J Physiol Gastrointest Liver Physiol 290: G757–G764, 2006. doi:10.1152/ajpgi.00408.2005. 16269520

[51] Viscardi RM, Lyon NH, Sun CCJ, Hebel JR, Hasday JD. Inflammatory cytokine mRNAs in surgical specimens of necrotizing enterocolitis and normal newborn intestine. Pediatr Pathol Lab Med 17: 547–559, 1997. doi:10.1080/15513819709168731. 9211547

[52] Hackam DJ, Good M, Sodhi CP. Mechanisms of gut barrier failure in the pathogenesis of necrotizing enterocolitis: toll-like receptors throw the switch. Semin Pediatr Surg 22: 76–82, 2013. doi:10.1053/j.sempedsurg.2013.01.003. 23611610 PMC3644853

[53] Snyder KB, Hunter CJ. The leaky gut: a narrative review on the role of epithelial cell permeability in necrotizing enterocolitis. Pediatr Med 7: 20–20, 2024. doi:10.21037/pm-22-41.

[54] Guerville M, Leroy A, Sinquin A, Laugerette F, Michalski MC, Boudry G. Western-diet consumption induces alteration of barrier function mechanisms in the ileum that correlates with metabolic endotoxemia in rats. Am J Physiol Endocrinol Physiol 313: E107–E120, 2017. doi:10.1152/ajpendo.00372.2016. 28400412

[55] Volynets V, Louis S, Pretz D, Lang L, Ostaff MJ, Wehkamp J, Bischoff SC. Intestinal barrier function and the gut microbiome are differentially affected in mice fed a Western-style diet or drinking water supplemented with fructose. J Nutr 147: 770–780, 2017. doi:10.3945/jn.116.242859. 28356436

[56] van Roy F, Berx G. The cell-cell adhesion molecule E-cadherin. Cell Mol Life Sci 65: 3756–3788, 2008. doi:10.1007/s00018-008-8281-1. 18726070 PMC11131785

[57] Bryant DM, Stow JL. The ins and outs of E-cadherin trafficking. Trends Cell Biol 14: 427–434, 2004. doi:10.1016/j.tcb.2004.07.007. 15308209

[58] de Beco S, Amblard F, Coscoy S. New insights into the regulation of e-cadherin distribution by endocytosis. Int Rev Cell Mol Biol 295: 63–108, 2012. doi:10.1016/B978-0-12-394306-4.00008-3. 22449487

[59] Mehta S, Nijhuis A, Kumagai T, Lindsay J, Silver A. Defects in the adherens junction complex (E-cadherin/β-catenin) in inflammatory bowel disease. Cell Tissue Res 360: 749–760, 2015. doi:10.1007/s00441-014-1994-6. 25238996

[60] Talavera D, Castillo AM, Dominguez MC, Gutierrez AE, Meza I. IL8 release, tight junction and cytoskeleton dynamic reorganization conducive to permeability increase are induced by dengue virus infection of microvascular endothelial monolayers. J Gen Virol 85: 1801–1813, 2004. doi:10.1099/vir.0.19652-0. 15218164

[61] McNamara BP, Koutsouris A, O’Connell CB, Nougayréde JP, Donnenberg MS, Hecht G. Translocated EspF protein from enteropathogenic Escherichia coli disrupts host intestinal barrier function. J Clin Invest 107: 621–629, 2001. doi:10.1172/JCI11138. 11238563 PMC199424

[62] Harhaj NS, Barber AJ, Antonetti DA. Platelet-derived growth factor mediates tight junction redistribution and increases permeability in MDCK cells. J Cell Physiol 193: 349–364, 2002. doi:10.1002/jcp.10183. 12384987

[63] Rao RK, Basuroy S, Rao VU, Karnaky KJ Jr, Gupta A. Tyrosine phosphorylation and dissociation of occludin–ZO-1 and E-cadherin–β-catenin complexes from the cytoskeleton by oxidative stress. Biochem J 368: 471–481, 2002. doi:10.1042/bj20011804. 12169098 PMC1222996

[64] Ivanov AI, Nusrat A, Parkos CA. Endocytosis of epithelial apical junctional proteins by a clathrin-mediated pathway into a unique storage compartment. Mol Biol Cell 15: 176–188, 2004. doi:10.1091/mbc.e03-05-0319. 14528017 PMC307538

[65] Buonpane C, Yuan C, Wood D, Ares G, Klonoski SC, Hunter CJ. ROCK1 inhibitor stabilizes E-cadherin and improves barrier function in experimental necrotizing enterocolitis. Am J Physiol Gastrointest Liver Physiol 318: G781–G792, 2020. doi:10.1152/ajpgi.00195.2019. 32090605 PMC7191467

[66] Ivanov AI, Nusrat A, Parkos CA. The epithelium in inflammatory bowel disease: potential role of endocytosis of junctional proteins in barrier disruption. inflammatory bowel disease. Novartis Found Symp 263: 115–132, 2004. 15669638

[67] Bhat AA, Syed N, Therachiyil L, Nisar S, Hashem S, Macha MA, Yadav SK, Krishnankutty R, Muralitharan S, Al-Naemi H, Bagga P, Reddy R, Dhawan P, Akobeng A, Uddin S, Frenneaux MP, El-Rifai W, Haris M. Claudin-1, a double-edged sword in cancer. Int J Mol Sci 21: 569, 2020. doi:10.3390/ijms21020569. 31952355 PMC7013445

[68] Bruewer M, Luegering A, Kucharzik T, Parkos CA, Madara JL, Hopkins AM, Nusrat A. Proinflammatory cytokines disrupt epithelial barrier function by apoptosis-independent mechanisms. J Immunol 171: 6164–6172, 2003. doi:10.4049/jimmunol.171.11.6164. 14634132

[69] Han X, Fink MP, Delude RL. Proinflammatory cytokines cause no·- dependent and -independent changes in expression and localization of tight junction proteins in intestinal epithelial cells. Shock 19: 229–237, 2003. doi:10.1097/00024382-200303000-00006. 12630522

[70] Liu D, Xu Y, Feng J, Yu J, Huang J, Li Z. Mucins and tight junctions are severely altered in necrotizing enterocolitis neonates. Am J Perinatol 38: 1174–1180, 2021. doi:10.1055/s-0040-1710558. 32446259

[71] Tso P, Balint JA. Formation and transport of chylomicrons by enterocytes to the lymphatics. Am J Physiol 250: G715–G726, 1986. doi:10.1152/ajpgi.1986.250.6.G715. 3521320

[72] Jaquez-Durán G, Arellano-Ortiz AL. Western diet components that increase intestinal permeability with implications on health: Hogrefe AG. Int J Vitam Nutr Res 94: 405–421, 2024. doi:10.1024/0300-9831/a000801. 38009780

[73] Ghoshal S, Witta J, Zhong J, de Villiers W, Eckhardt E. Chylomicrons promote intestinal absorption of lipopolysaccharides. J Lipid Res 50: 90–97, 2009. doi:10.1194/jlr.M800156-JLR200. 18815435

[74] Jerala R Structural biology of the LPS recognition. Int J Med Microbiol 297: 353–363, 2007. doi:10.1016/j.ijmm.2007.04.001. 17481951

[75] Chan KL, Wong KF, Luk JM. Role of LPS/CD14/TLR4-mediated inflammation in necrotizing enterocolitis: pathogenesis and therapeutic implications. World J Gastroenterol 15: 4745–4752, 2009. doi:10.3748/wjg.15.4745. 19824106 PMC2761550

[76] Candelli M, Franza L, Pignataro G, Ojetti V, Covino M, Piccioni A, Gasbarrini A, Franceschi F. Interaction between lipopolysaccharide and gut microbiota in inflammatory bowel diseases. Int J Mol Sci 22: 6242, 2021. doi:10.3390/ijms22126242. 34200555 PMC8226948

[77] Kelman Z PCNA: structure, functions and interactions. Oncogene. 14: 629–640, 1997. doi:10.1038/sj.onc.1200886. 9038370

[78] Toschi L, Bravo R. Changes in cyclin/proliferating cell nuclear antigen distribution during DNA repair synthesis. J Cell Biol 107: 1623–1628, 1988. doi:10.1083/jcb.107.5.1623. 2903166 PMC2115310

[79] Conlon MA, Kerr CA, McSweeney CS, Dunne RA, Shaw JM, Kang S, Bird AR, Morell MK, Lockett TJ, Molloy PL, Regina A, Toden S, Clarke JM, Topping DL. Resistant starches protect against colonic DNA damage and alter microbiota and gene expression in rats fed a Western diet. J Nutr 142: 832–840, 2012. doi:10.3945/jn.111.147660. 22457395 PMC3327741

[80] Wang L, Li J, Li Q, Zhang J, Duan XL. Morphological changes of cell proliferation and apoptosis in rat jejunal mucosa at different ages. World J Gastroenterol 9: 2060–2064, 2003. doi:10.3748/wjg.v9.i9.2060. 12970906 PMC4656674

[81] Uxa S, Castillo-Binder P, Kohler R, Stangner K, Müller GA, Engeland K. Ki-67 gene expression. Cell Death Differ 28: 3357–3370, 2021. doi:10.1038/s41418-021-00823-x. 34183782 PMC8629999

[82] Doganay S, Budak O, Bahtiyar N, Toprak V. Effects of ketogenic and Western diets on proliferation, vasculogenesis and oxidative stress in the liver. JECM 38: 312–317, 2021. doi:10.52142/omujecm.38.3.20.

[83] Kullmann F, Fadaie M, Gross V, Knüchel R, Bocker T, Steinbach P, Schölmerich J, Rüschoff J. Expression of proliferating cell nuclear antigen (PCNA) and Ki-67 in dysplasia in inflammatory bowel disease. Eur J Gastroenterol Hepatol 8: 371–379, 1996. doi:10.1097/00042737-199604000-00016. 8781908

[84] Neavin DR, Liu D, Ray B, Weinshilboum RM. The role of the aryl hydrocarbon receptor (AHR) in immune and inflammatory diseases. Int J Mol Sci 19: 3851, 2018. doi:10.3390/ijms19123851. 30513921 PMC6321643

[85] Catrysse L, Vereecke L, Beyaert R, van Loo G. A20 in inflammation and autoimmunity. Trends Immunol 35: 22–31, 2014. doi:10.1016/j.it.2013.10.005. 24246475

[86] Rogero MM, Calder PC. Obesity, inflammation, toll-like receptor 4 and fatty acids. Nutrients. 10: 432, 2018. doi:10.3390/nu10040432. 29601492 PMC5946217

[87] Golubkova A, Leiva T, Snyder K, Schlegel C, Bonvicino SM, Agbaga M-P, Brush RS, Hansen JM, Vitiello PF, Hunter CJ. Response of the glutathione (GSH) antioxidant defense system to oxidative injury in necrotizing enterocolitis. Antioxidants (Basel) 12: 1385, 2023. doi:10.3390/antiox12071385. 37507924 PMC10376622

